# Clinical Effects of a Digital Health Intervention for Adults With Type 2 Diabetes in the United States: Retrospective Cohort Study

**DOI:** 10.2196/66911

**Published:** 2026-06-09

**Authors:** David Kerr, Nita Thingalaya, Praveen Potukuchi, Keni CS Lee, Edward Jonathan Han-Burgess, Darren Frey, Alison Edwards, Xinyan Yu, Laura Wilson, Felix Lee

**Affiliations:** 1 Center for Health Systems Research Sutter Health Santa Barbara, CA United States; 2 Sanofi Morristown, NJ United States; 3 Sanofi Reading, Berkshire United Kingdom; 4 Sanofi Cambridge, MA United States; 5 Ensemble Insight Paris France; 6 Sciences Po Paris France; 7 Symphony Health ICON plc Blue Bell, PA United States

**Keywords:** digital health, type 2 diabetes mellitus, T2DM, glycated hemoglobin, HbA_1c_, severe hypoglycemia, self-management, medication adherence, glycemic control, diabetic comprehensive care, diabetes, cohort, diabetes mellitus, blood glucose, BG, glucose, adult, adults, antidiabetic medication, antidiabetic medications, glycemia

## Abstract

**Background:**

Digital health interventions show promise in supporting adults with type 2 diabetes mellitus (T2DM). Dario is a digital diabetes solution (DDS) that allows individuals with T2DM to automatically log blood glucose (BG) measurements and other variables to facilitate positive behavior changes.

**Objective:**

This retrospective cohort study compared real-world clinical outcomes of adults with T2DM using the DDS with those of adults with T2DM receiving usual care alone.

**Methods:**

Health care and claims data collected from the DDS, the Symphony Health Integrated Dataverse, and the Quest Laboratory were integrated into a single dataset using tokenization. DDS users were compared with a matched cohort of nonusers over the period from January 2017 to October 2021. Inclusion criteria were adults ≥18 years of age with T2DM, treated with antidiabetic medications, with ≥7% glycated hemoglobin (HbA_1c_) prior to a defined index date, and with valid HbA_1c_ measurements during follow-up. DDS users were matched 1:3 with nonusers using exact-matching criteria and propensity score matching. Difference-in-difference values were determined using a multivariable linear regression model. The primary endpoint was change in HbA_1c_ from baseline to 6 months.

**Results:**

The two cohorts included 568 DDS users and 1699 nonusers with matched baseline characteristics. At 6 months, the generalized linear model least squares mean estimate of the difference in change in HbA_1c_ between the two cohorts was −0.23% (95% CI −0.38% to −0.07%, *P*=.004). Similarly, HbA_1c_ decreased significantly more in DDS users versus nonusers in sensitivity analyses at 12 months. In four subgroups of individuals with baseline HbA_1c_>7.5%, >8.0%, >9.0%, and >11.0%, HbA_1c_ decreased significantly more in DDS users than in nonusers at 6 and 12 months. At 6 months, 47.0% of DDS users and 36.8% of nonusers had a ≥1% decrease in HbA_1c_ from baseline (*P*<.001). Rates of severe hypoglycemia did not increase in either cohort between baseline and 6 months. At 6 months, more DDS users with baseline HbA_1c_≥8% had achieved HbA_1c_<8% (*P*=.002). At 6-month follow-up, medication adherence rates were evaluated as an exploratory endpoint and found to be greater for DDS users than for nonusers (*P*=.03). Engagement with the DDS was also evaluated as an exploratory endpoint and was associated with a significant decrease in HbA_1c_ from baseline to follow-up (r=−0.28, *P*<.001). Engagement activities associated with reduced HbA_1c_ were BG measurement and tagging (timing of BG measurement and meal type).

**Conclusions:**

Adults with T2DM and elevated HbA_1c_ levels using the DDS had better outcomes at 6 months than matched nonusers across various baseline HbA_1c_ levels, with no increase in the rates of severe hypoglycemia. These effects were sustained at 12 months. The results confirm that digital interventions that facilitate behavior change can potentially deliver improvements in health outcomes.

## Introduction

In type 2 diabetes mellitus (T2DM), poor glycemic control is associated with an increased incidence of microvascular and macrovascular complications and reduced quality of life. However, many people with T2DM find it difficult to achieve their individualized glycemic targets [1]. Furthermore, therapeutic inertia is common, leading to sustained hyperglycemia [1,2]. Therapeutic inertia is a multifaceted issue that is influenced by many factors, including lack of knowledge, poor medication adherence, and psychological factors [1,2]. Measures to improve glycated hemoglobin (HbA_1c_) in people with T2DM are therefore needed, and self-management education and structured self-monitoring of blood glucose (BG) have been shown to be effective strategies [3].

Digital health interventions are becoming increasingly available and show promise in supporting the self-management of diabetes [4-6]. For any digital health technology to be accepted by health care providers and payers in the United States, meaningful and reliable outcomes data are crucial. Few rigorous comparisons to quantify the benefits of digital health interventions have been published, and the studies performed so far have often been of poor quality [7]. Further, digital therapeutics that have been authorized by the US Food and Drug Administration and require a clinician’s prescription also have important limitations in the rigor of the underlying evidence [8].

Dario, a digital diabetes solution (DDS), is a nonprescription digital health intervention with a smartphone app that aims to facilitate positive behavior changes leading to improved diabetes outcomes (Multimedia Appendix 1) [9]. The DDS allows the user to monitor BG levels and to log meals, carbohydrate consumption, insulin intake, physical activity, and other parameters through the app interface, as well as generate reports that can be shared with health care providers. The aim of this retrospective study was to compare real-world clinical outcomes of adults with T2DM using the DDS with those not using the DDS.

## Methods

### Study Design and Data Sources

**Figure 1:**
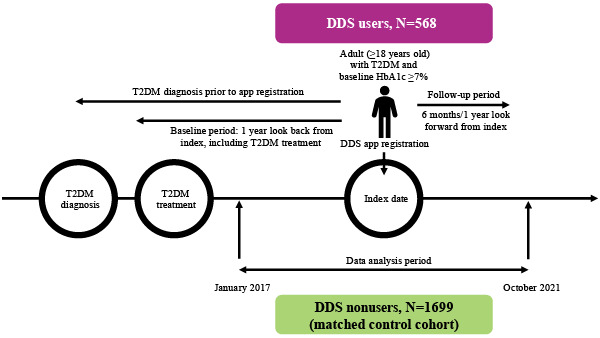
Overview of the retrospective cohort study design. DDS: digital diabetes solution; HbA_1c_: glycated hemoglobin; T2DM: type 2 diabetes mellitus.

An abstract describing this study has been published earlier [10], and additional methodological details are included in Multimedia Appendix 2. Briefly, this was a retrospective cohort study in which adults with T2DM who had used the Dario DDS from January 1, 2017, to October 31, 2021, were matched with individuals with T2DM who had not used the Dario DDS in the same period. DDS nonusers were randomly assigned to a single quarter in which they had a medical claim for any diagnosis. An index date was defined as the first registration for DDS users or the first claim date in the quarter for nonusers. The follow-up period for the primary and secondary endpoints was 6 months. Some endpoints were also evaluated at 12 months (Figure 1).

The study used routinely collected real-world data from the DDS, the Symphony Health Integrated Dataverse (IDV), and the Quest Laboratory. The IDV is a database that represents approximately 286 million active patients and includes claims data linked to electronic medical records (EMR) [11]. The study used US pharmacy/medical claims data and EMR data from the IDV. HbA_1c_ results were retrieved from Quest Laboratory data [12] as the primary source, supplemented by EMR, where appropriate. Data from DDS users were deidentified and tokenized with Synoma ID so that the records from DDS, IDV, and Quest data could be triple-linked.

### Study Population

Inclusion criteria were as follows: adults ≥18 years of age with T2DM; treated with any antidiabetic medications before the index date; with ≥1 valid HbA_1c_ measurements during the baseline period; with the most recent preindex HbA_1c_≥7%; with ≥1 valid HbA_1c_ measurements from index date + 31 days to index date + 420 days; and with ≥90 days between baseline and postindex periods. Patients were excluded if they were <18 years old, were using continuous glucose monitoring, or were diagnosed with type 1 diabetes or gestational diabetes.

The DDS cohort consisted of all DDS users who satisfied all the inclusion criteria. The DDS nonuser cohort was selected from the universe of IDV patients meeting all the inclusion criteria and none of the exclusion criteria. Sequential 1:3 exact and propensity score matching (PSM) methods were applied to obtain the study cohort. Sex, payer type, quarter in which the index date fell, and medication type (oral antidiabetic drug [OAD], insulin, any other injectable antidiabetic drug, or any combination) were used for exact matching. Covariates used for PSM additionally included baseline HbA_1c_, age, race/ethnicity, region, Charlson comorbidity score, comorbidities (hypertension, hyperlipidemia, anemia, depression), and comedications (concomitant medications that could impact BG levels [eg, steroids]).

### Endpoints

The primary endpoint was change in HbA_1c_ from baseline to 6 months. Over the same period, secondary endpoints were change in the severe hypoglycemia event rate from baseline, percentage of patients achieving the HbA_1c_ target of <8% [13], and change in the severe hypoglycemia event rate in patients with HbA_1c_ <8%. Severe hypoglycemia events were defined using the *International Classification of Diseases* (ICD)-9 or ICD-10 diagnosis codes from medical claims.

Exploratory endpoints included change in medication adherence and association with HbA_1c_ change and the impact of the engagement activity frequency in DDS users on achieved HbA_1c_ levels, both at 6 months. For DDS users, DDS engagement frequency was defined by the number of active days within the 180‑day postindex period. An active day was defined as one in which an individual performed one of the following 10 activities: measuring BG, measuring blood pressure (BP), measuring weight, tagging (timing of BG measurement and meal type), food logging (carbohydrate counting, meal photos, etc), recording the insulin dose, recording physical activity, sharing the logbook, reading an educational article, and interacting with a coach. The change in diabetic comprehensive care measures for DDS users and nonusers at 12 months was also measured. To align with HEDIS (Healthcare Effectiveness Data and Information Set) quality standards, these care measures were defined as the percentage of patients with the following details: HbA_1c_>9%, HbA_1c_<8%, nephropathy monitoring, retinopathy exam, and BP control<140/90 mm Hg [14].

Further exploratory endpoints were the difference between DDS users and nonusers in changes in the following parameters at 6 months, extracted from EMR data: fasting BG; total cholesterol, triglycerides, and low-density lipoprotein cholesterol (LDL-c); estimated glomerular filtration rate (eGFR); mean systolic blood pressure (SBP) and diastolic blood pressure (DBP); body weight/BMI; and cardiovascular disease (CVD) risk, as measured using the Framingham risk score [15]. Change in the CVD risk score was also measured at 12 months.

Primary and secondary endpoints were analyzed at 12-month follow-up as a sensitivity analysis. Subgroup analyses were conducted for the primary and secondary endpoints in various groups according to HbA_1c_ values, age, the presence/absence of severe hypoglycemia, and antidiabetic treatment.

### Statistical Methods

It was calculated that 513 individuals were needed in the DDS user cohort and 1539 matched DDS nonusers to achieve 80% power with α=.05 (see Multimedia Appendix 2 for full details).

Continuous variables were statistically compared between the DDS user and nonuser cohorts using *t* tests if the assumption of normality held or the Wilcoxon rank-sum test if a nonparametric test was needed based on a review of histograms. For categorical variables, study cohorts were compared using chi-square tests or Fisher’s exact test, whichever was appropriate, given cell sizes and the number of categories. Multivariable linear regression with a fixed-effect model (generalized linear model [GLM]) was generated to determine the association between DDS users and follow-up HbA_1c_ compared with DDS nonusers. For endpoints reported as changes from baseline to follow-up, a difference-in-difference (D-i-D) method was used and least squares means were estimated with 95% CIs.

Medication adherence was measured by drug class using the proportion of days covered (PDC) over the 6 months preceding and 6 months following the index date. Baseline and follow-up measures of the PDC were compared as a continuous variable between the two cohorts. For evaluation of the impact of the engagement activity frequency in DDS users on achieved HbA_1c_ levels, collinearity was evaluated by determining variance inflation factors (VIFs).

### Ethical Considerations

This observational study was performed in accordance with ethical principles consistent with the Declaration of Helsinki, the International Council for Harmonisation of Technical Requirements for Pharmaceuticals for Human Use Guideline for Good Clinical Practice, Good Pharmacoepidemiology Practices, and the applicable legislation on noninterventional studies and/or observational studies. The investigators performed the observational study in accordance with the regulations and guidelines governing medical practice and ethics in the United States and in accordance with currently acceptable techniques and know-how. Human subjects were not affected by the study as it was a retrospective, anonymized data analysis. The study was reviewed by the Salus Institutional Review Board and granted a waiver of informed consent (IRB #23538).

By providing sensitive personal information (including health information and protected health information), users of the Dario DDS explicitly consented to the collection, use, and sharing of their sensitive personal information in accordance with the privacy policy [16]. In line with the privacy policy consented to by the users upon registering for an account, once personal information is aggregated, deidentified, and/or anonymized, it is no longer considered personal. Therefore, continued use of the Dario DDS is contingent upon this consent, and users do not have a specific option to opt out of their deidentified data being used for research purposes.

## Results

### Demographic and Baseline Characteristics

**Figure 2:**
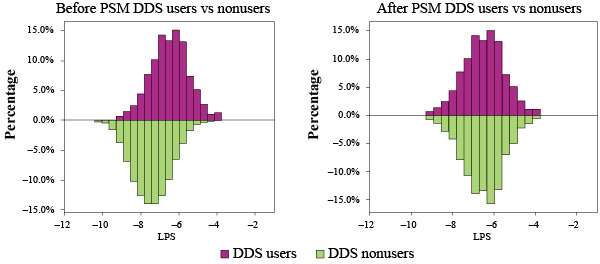
Mirrored histogram of propensity scores. DDS: digital diabetes solution; LPS: longest prefix suffix; PSM: propensity score matching.

The matched cohorts included 568 Dario DDS users and 1699 nonusers. Propensity scores for DDS users and nonusers, before and after PSM, are shown in Figure 2. Demographic and baseline characteristics were similar between the two groups (Table 1).

**Table 1:** Demographic and baseline characteristics of cohorts after matching.

Characteristics		DDS^a^ users (N=568)	DDS nonusers (N=1699)
**Sex,^b^** **n (%)**			
	Female	262 (46.1)	784 (46.1)
	Male	306 (53.9)	915 (53.9)
Age^b^ (years), mean (SD)		57.3 (10.5)	57.6 (11.6)
**Age group (years), n (%)**			
	18-25	0	12 (0.7)
	26-35	14 (2.5)	44 (2.6)
	36-45	61 (10.7)	191 (11.2)
	46-55	164 (28.9)	445 (26.2)
	56-65	202 (35.6)	624 (36.7)
	66-75	103 (18.1)	278 (16.4)
	≥76	24 (4.2)	105 (6.2)
**Race/ethnicity, n (%)**			
	African American	55 (9.7)	173 (10.2)
	Asian	8 (1.4)	22 (1.3)
	White	316 (55.6)	933 (54.9)
	Hispanic	66 (11.6)	202 (11.9)
	Other	10 (1.7)	31 (1.8)
	Unknown	113 (19.9)	338 (19.9)
**Geographic region, n (%)**			
	Mid-Atlantic	95 (16.7)	271 (16.0)
	Midwest	79 (13.9)	230 (13.5)
	Northeast	27 (4.8)	87 (5.1)
	Northwest	10 (1.8)	29 (1.7)
	Other territories	3 (0.5)	10 (0.6)
	Southeast	185 (32.6)	555 (32.7)
	Southwest	100 (17.6)	331 (19.5)
	West	69 (12.2)	186 (11.0)
**Payer type, n (%)**			
	Cash	3 (0.5)	9 (0.5)
	Commercial	398 (70.1)	1190 (70.0)
	Medicaid/managed Medicaid	28 (4.9)	84 (4.9)
	Medicare	101 (17.8)	303 (17.8)
	Other^c^	38 (6.7)	113 (6.7)
**Index year, n (%)**			
	2017	115 (20.3)	345 (20.3)
	2018	121 (21.3)	363 (21.4)
	2019	103 (18.1)	308 (18.1)
	2020	125 (22.0)	374 (22.0)
	2021^d^	104 (18.3)	309 (18.2)
**HbA_1c_^a,e^** **(%)**			
	Mean (SD)	9.14 (1.78)	9.13 (1.85)
	Median (minimum-maximum)	8.6 (7-15.1)	8.6 (7-16.5)
	HbA_1c_≥8%, n (%)	387 (68.1)	1089 (64.1)
	HbA_1c_>9%, n (%)	237 (41.7)	713 (42.0)
**Antidiabetic medication, n (%)**			
	Insulin only	34 (6.0)	101 (5.9)
	OAD^f^ only	289 (50.9)	864 (50.9)
	Any combination/other injectable	245 (43.1)	734 (43.2)
**Charlson comorbidity index score**			
	Mean (SD)	1.5 (1.5)	1.6 (1.6)
	Median (minimum-maximum)	1 (0-9)	1 (0-13)

^a^DDS: digital diabetes solution.

^b^Standardized mean differences for sex, age, and baseline HbA_1c_ are reported in Multimedia Appendix 3.

^c^Assistance programs and/or coupons.

^d^2021 was a partial year.

^e^HbA_1c_: glycated hemoglobin.

^f^OAD: oral antidiabetic drug.

### HbA_1c_ Change from Baseline to 6 and 12 Months

**Figure 3:**
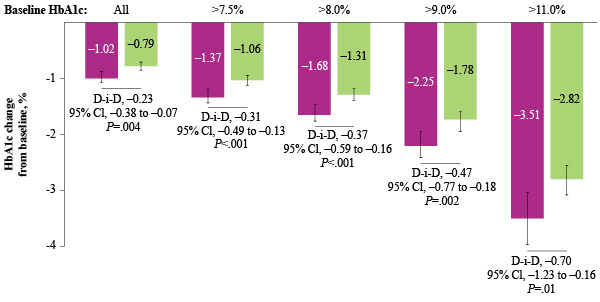
Mean change in HbA_1c_ from baseline to 6-month follow-up in all patients and in subgroups by baseline HbA_1c_. Bars represent the mean change from baseline. Error bars represent 95% CIs. DDS: digital diabetes solution; D-i-D: difference-in-difference; HbA_1c_: glycated hemoglobin.

Over 6 months, HbA_1c_ for the DDS group fell significantly (−1.02%, 95% CI −1.15 to −0.89], *P*<.001) (Table 2 and Figure 3). In comparison, in the nonuser cohort, HbA_1c_ fell by 0.79% (95% CI −0.87 to −0.71, *P*<.001). The GLM least squares mean estimate of the difference in change in HbA_1c_ between the two cohorts was −0.23% (95% CI −0.38% to −0.07%, *P*=.004).

The change in mean HbA_1c_ was statistically significantly greater among DDS users than among nonusers at 12 months (*P*=.03). See Multimedia Appendix 4 for details.

**Table 2:** Mean change in HbA_1c_^a^ from baseline to 6 months in all patients and in subgroups by baseline HbA_1c_.

Endpoints			Baseline				Follow-up				D-i-D^c^		*P* value
			DDS^b^ users (N=568)		DDS nonusers (N=1699)		DDS users (N=568)		DDS nonusers (N=1699)				
**Primary endpoint: all**													
	Patients, n (%)	568 (100.0)		1699 (100.0)		568 (100.0)		1699 (100.0)		—^d^		—	
	HbA_1c_, mean (SD)	9.14 (1.78)		9.13 (1.85)		8.12 (1.69)		8.34 (1.78)		—		—	
	Change from baseline (GLM^e^), mean (95% CI)	—		—		−1.02 (−1.15 to −0.89)		−0.79 (−0.87 to −0.71)		−0.23 (−0.38 to −0.07)		.004^f^	
	*P* value	—		—		<.001		<.001		—		—	
**Subgroup analysis: HbA_1c_>7.5% at baseline**													
	Patients, n (%)	459 (80.8)		1323 (77.9)		459 (80.8)		1323 (77.9)		—		—	
	HbA_1c_, mean (SD)	9.58 (1.70)		9.67 (1.76)		8.26 (1.72)		8.60 (1.85)		—		—	
	Change from baseline (GLM), mean (95% CI)	—		—		−1.37 (−1.52 to −1.21)		−1.06 (−1.15 to −0.96)		−0.31 (−0.49 to −0.13)		<.001^f^	
	*P* value	—		—		<.001		<.001		—		—	
**Subgroup analysis: HbA_1c_>8.0% at baseline**													
	Patients, n (%)	374 (65.8)		1044 (61.4)		374 (65.8)		1044 (61.4)		—		—	
	HbA_1c_, mean (SD)	9.99 (1.63)		10.18 (1.64)		8.40 (1.76)		8.83 (1.92)					
	Change from baseline (GLM), mean (95% CI)	—		—		−1.68 (−1.87 to −1.50)		−1.31 (−1.42 to −1.20)		­0.37 (−0.59 to −0.16)		<.001^f^	
	*P* value	—		—		<.001		<.001		—		—	
**Subgroup analysis: HbA_1c_>9.0% at baseline**													
	Patients, n (%)	237 (41.7)		713 (42.0)		237 (41.7)		713 (42.0)		—		—	
	HbA_1c_, mean (SD)	10.85 (1.45)		10.96 (1.41)		8.66 (1.91)		9.16 (2.06)		—		—	
	Change from baseline (GLM), mean (95% CI)	—		—		−2.25 (−2.50 to −1.99)		−1.78 (−1.92 to −1.63)		−0.47 (−0.77 to −0.18)		.002^f^	
	*P* value	—		—		<.001		<.001		—		—	
**Subgroup analysis: HbA_1c_>11.0% at baseline**													
	Patients, n (%)	92 (16.2)		291 (17.1)		92 (16.2)		291 (17.1)		—		—	
	HbA_1c_, mean (SD)	12.43 (0.93)		12.38 (0.99)		8.88 (2.25)		9.57 (2.3)		—		—	
	Change from baseline (GLM), mean (95% CI)	—		—		−3.51 (−3.98 to −3.04)		−2.82 (−3.08 to −2.55)		−0.70 (−1.23 to −0.16)		.01^f^	
	*P* value	—		—		<.001		<.001		—		—	

^a^HbA_1c_: glycated hemoglobin.

^b^DDS: digital diabetes solution.

^c^D-i-D: difference-in-difference.

^d^Not applicable.

^e^GLM: generalized linear model.

^f^Significant *P* values.

### Subgroup Analyses

Analyses were conducted for four subgroups of patients based on their baseline HbA_1c_ (>7.5%, >8.0%, >9.0%, and >11.0%). In each subgroup, HbA_1c_ decreased significantly more in DDS users than in nonusers at 6 months (Table 2 and Figure 3). Similarly, in the sensitivity analyses, HbA_1c_ decreased significantly more in DDS users than in nonusers in each subgroup at 12 months (Multimedia Appendix 4). In both groups, absolute reductions in HbA_1c_ were greater in patients with higher baseline HbA_1c_ levels.

The percentage of patients who experienced an absolute HbA_1c_ decrease of ≥1% from baseline was significantly greater among DDS users compared with nonusers at 6 months (Multimedia Appendix 5). The results did not differ significantly between the groups at 12 months.

The absolute HbA_1c_ change from baseline was compared for DDS users and nonusers at 6 and 12 months in the following subgroups: patients aged ≥65 years, patients on insulin with or without other antidiabetic drugs, and patients on antidiabetic drugs other than insulin. Between-group differences in these subgroups were not statistically significant (data not shown), except for patients on antidiabetic drugs other than insulin. In this subgroup, DDS users were significantly more successful than nonusers at 6 months, with a D-i-D of −0.31% (95% CI −0.5% to −0.1%, *P*=.001). See Multimedia Appendix 5 for details.

To examine the effect of any potential between-group behavioral differences, use of test strips was considered as a proxy for the level of self-motivation in diabetes care. A subgroup analysis was performed on DDS users and nonusers who were using test strips. The decrease in HbA_1c_ did not differ significantly between the two groups at 6 or 12 months (Multimedia Appendix 5).

### Secondary Endpoints

The rate of severe hypoglycemia in DDS users and nonusers did not increase between baseline and 6 months. Using the GLM, the estimated mean rate of events per 6 months was 0.0024 among DDS users and 0.0027 among nonusers (*P*=.93). See Table 3 for details.

At 6 months, 174/387 (45.0%) DDS users and 393/1089 (36.1%) nonusers who had a baseline HbA_1c_ of ≥8% had achieved an HbA_1c_ target of <8% (*P*=.002).

The difference in the change in the severe hypoglycemia event rate from baseline to 6-month follow-up between DDS users and nonusers was nonsignificant for the entire cohort and in a subgroup of patients with HbA_1c_<8% at baseline (Table 3). Similar results were observed at 12 months (Table 3).

At 12 months, the percentage of patients who achieved HbA_1c_<8% did not differ between DDS users and nonusers with severe hypoglycemia or without severe hypoglycemia (Multimedia Appendix 5).

**Table 3:** Change in the severe hypoglycemia event rate from baseline to 6 months in all patients and in patients with HbA_1c_^a^<8%.

Endpoints			Baseline				Follow-up				Incidence rate ratio		*P* value
			DDS^b^ users (N=568)		DDS nonusers (N=1699)		DDS users (N=568)		DDS nonusers (N=1699)				
**All patients**													
	Patients, n (%)	568 (100.0)		1699 (100.0)		568 (100.0)		1699 (100.0)		—^c^		—	
	Patients with severe hypoglycemia, n (%)	7 (1.2)		12 (0.7)		4 (0.7)		6 (0.4)		—		—	
	Severe hypoglycemia events, n	9		18		11		9		—		—	
	Severe hypoglycemia events, mean (SD)	0.02 (0.15)		0.01 (0.15)		0.02 (0.26)		0.01 (0.11)		—		—	
	Follow-up severe hypoglycemia (GLM^d,e^), mean events per 6 months (95% CI)	—		—		0.0024 (0.0003-0.0176)		0.0027 (0.0010-0.0071)		0.91 (0.09-8.76)		.93	
**HbA_1c_<8.0%**													
	Patients with baseline HbA_1c_≥8% reaching HbA_1c_<8%, n (%)	174 (30.6)		393 (69.2)		174 (30.6)		393 (69.2)		—		—	
	Patients with severe hypoglycemia, n (%)	2 (1.1)		5 (1.3)		1 (0.6)		3 (0.8)		—		—	
	Severe hypoglycemia events, n	3		7		3		6		—		—	
	Severe hypoglycemia events, mean (SD)	0.02 (0.17)		0.02 (0.18)		0.02 (0.23)		0.02 (0.21)		—		—	
	Follow-up severe hypoglycemia (GLM^e^), mean events per 6 months (95% CI)	—		—		0.0045 (0.0003-0.0672)		0.0044 (0.0009-0.0212)		1.01 (0.03-25.47)		.99	

^a^HbA_1c_: glycated hemoglobin.

^b^DDS: digital diabetes solution.

^c^Not applicable.

^d^GLM: generalized linear model.

^e^GLM-specified count of severe hypoglycemia events as following a negative binomial distribution.

### Exploratory Endpoints

#### Change in Medication Adherence in Association With HbA_1c_ Change

Adherence rates during the 6-month follow-up period are shown in Table 4 for individuals with medication adherence data available. The D-i-D between the groups for the percentage of patients with ≥80% PDC was 2.16% (95% CI 0.2% to 4.1%, *P*=.03) in favor of the DDS user group.

During the 6-month follow-up period, among individuals with medication adherence data available, the PDC was similar in both groups (*P*=.31), as shown in Table 4. In the follow-up period, the overall model (controlling for baseline medication class and follow-up PDC) showed that patients with ≥80% adherence experienced on average 0.36% more HbA_1c_ reduction than patients with <60% adherence (*P*=.01). DDS users had 0.28% more HbA_1c_ reduction than nonusers (*P*=.006). See Multimedia Appendix 6 for details. When controlling for the follow-up PDC, the reduction in HbA_1c_ did not differ between DDS users and nonusers by medication class, except among patients on combination treatment (Multimedia Appendix 6).

**Table 4:** Adherence rates and change in adherence among DDS^a^ users and nonusers.

Endpoints	Baseline		Follow-up		D-i-D^b^	*P* value
	DDS users (N=568)	DDS nonusers (N=1699)	DDS users (N=568)	DDS nonusers (N=1699)		
Patients with adherence data available,^c^ n (%)	498 (87.7)	1494 (87.9)	498 (87.7)	1494 (87.9)	—^d^	—
Patients with ≥80% PDC^c,e^ in follow-up period, n (%)	—	—	391 (78.5)	1140 (76.3)	—	.31
PDC (%), mean (SD)	76.5 (30.1)	78.3 (28.3)	88.1 (19.4)	86.3 (20.7)	—	—
Change from baseline (GLM^f^ %), mean (95% CI)	—	—	10.6 (8.9-12.3)	8.4 (7.4-9.4)	2.1 (0.2-4.1)	.03^g^

^a^DDS: digital diabetes solution.

^b^D-i-D: difference-in-difference.

^c^Patients with antidiabetic treatment (oral antidiabetic drug [OAD], insulin, other injectable, or any combination) during baseline and follow-up periods.

^d^Not applicable.

^e^PDC: proportion of days covered.

^f^GLM: generalized linear model.

^g^Significant *P* values.

#### Change in HbA_1c_ by Engagement Activity in DDS Users

The mean overall DDS engagement activity was 75.4 (SD 55.9) days during the 180-day follow-up period. The median overall engagement activity was 64.5 (IQR 25-124) active days.

**Figure 4:**
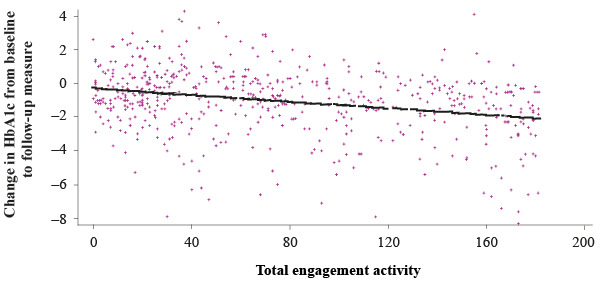
Correlation between overall DDS engagement activity and change in HbA_1c_, showing that greater engagement with the DDS was associated with a significant decrease in HbA_1c_ from baseline to follow-up (r=−0.28, *P*<.001). DDS: digital diabetes solution; HbA_1c_: glycated hemoglobin.

Descriptive statistics for overall and individual components of DDS engagement are provided in Table 5. Each day of engagement with any one of the 10 DDS engagement activities was associated with a 0.01% change in HbA_1c_ (*P*<.001). The correlation between overall DDS engagement activity and change in HbA_1c_ is shown in Figure 4. Greater engagement with the DDS was associated with a significant decrease in HbA_1c_ from baseline to follow-up (r=−0.28, *P*<.001).

Individual engagement activities with significant associations with reduced HbA_1c_ were BG measurement and tagging (timing of BG measurement and meal type). Recording the insulin dose had a significant association with increased HbA_1c_ (Multimedia Appendix 7).

When collinearity was evaluated, VIF values for all included engagement activities were between 1 and 2 (data not shown). As VIF≥10 would suggest collinearity among variables included in the model, collinearity was excluded.

**Table 5:** Descriptive statistics of DDS^a^ engagement and associations with change in HbA_1c_^b^ during 6-month follow-up.

Component of engagement	Patients with ≥1 active day (N=568), n (%)	Active days, median (IQR)	Active days, mean (SD)	β-coefficient (*P* value)
Any	566 (99.6)	64.5 (25-124)	75.4 (55.9)	–0.0105 (<.001^c^)
Measuring BG^d^	557 (98.1)	63.5 (24-122)	74.5 (55.8)	–0.0086 (<.001^c^)
Tagging (timing of BG measurement and meal type)	450 (79.2)	9 (1-49)	33.9 (48.5)	–0.0046 (.007^c^)
Sharing the logbook	425 (74.8)	1 (0-1)	1.3 (2.5)	0.0015 (0.96)
Measuring weight	206 (36.3)	0 (0-1)	3.3 (14.9)	0.0021 (0.64)
Inputting the insulin dose	131 (23.1)	0	9.1 (29.6)	0.0050 (.04^c^)
Food logging (carbohydrate counting, meal photos, etc)	126 (22.2)	0	2.6 (13.4)	–0.0064 (.20)
Reading an article	110 (19.4)	0	1.5 (6.5)	0.0007 (.95)
Measuring BP^e^	50 (8.8)	0	3.6 (20.0)	–0.0020 (.59)
Recording physical activity	45 (7.9)	0	0.5 (4.2)	–0.0085 (.61)
Interacting with a coach	5 (0.9)	0	0.1 (0.7)	–0.0618 (.52)

^a^DDS: digital diabetes solution.

^b^HbA_1c_: glycated hemoglobin.

^c^Significant *P* values.

^d^BG: blood glucose.

^e^BP: blood pressure.

#### Change in Diabetic Comprehensive Care Measures at 12-month Follow-Up

Of the 387/568 (68.1%) DDS users and 1089/1699 (64.1%) nonusers who had HbA_1c_≥8% at baseline, 179 (46.3%) and 419 (38.5%), respectively, had HbA_1c_<8% at 12 months (*P*=.007). See Multimedia Appendix 8 for details.

Similarly, of the 237/568 (41.7%) DDS users and 713/1699 (42.0%) nonusers who had HbA_1c_>9% at baseline, 86 (36.3%) and 347 (48.7%), respectively, still had HbA_1c_>9% at 12 months (*P*<.001). See Multimedia Appendix 8 for details.

No statistically significant between-group differences were noted at 12 months for the percentage of patients with BP control, appropriate nephropathy monitoring, or retinopathy exam (Multimedia Appendix 8).

#### Change in Laboratory Values From Baseline

Changes in the following values were measured in each group at baseline and 6-month follow-up: average fasting BG; total cholesterol, triglycerides, and LDL-c; eGFR; mean SBP and DBP; and body weight/BMI (Multimedia Appendix 9). Change in the CVD risk score was measured at 12 months (Multimedia Appendix 10). No statistically significant differences were noted between groups in changes in any of these measures at follow-up.

## Discussion

### Principal Findings

This retrospective cohort study examined the effect of 6 months of use of the Dario DDS, a digital health intervention, by adults with T2DM. For DDS users, the decrease in HbA_1c_ between baseline and 6-month follow-up was significantly greater than in nonusers. Furthermore, this improved glycemic control did not increase users’ risk of experiencing severe hypoglycemic events.

From the study reported here, several points can be noted about the favorable effect of DDS use on glycemic control. The additional lowering of HbA_1c_ in patients with T2DM who used the DDS is relevant to real-world clinical practice, as good BG control is a core goal of T2DM disease management [13]. The effect was shown to be associated with a sustained clinical impact at 12 months, and improved glycemic control was observed at population as well as individual levels. Improvements were seen in exploratory HEDIS-defined measures of care quality: the number of patients with good glycemic control increased, while those with poor glycemic control decreased. Although the mean HbA_1c_ decrease for the population overall was small, greater reductions from subanalyses were seen in individuals with higher baseline HbA_1c_ levels.

The absolute HbA_1c_ change from baseline did not differ significantly between DDS users and nonusers at 6 and 12 months in subgroups including patients aged ≥65 years or insulin users with or without other antidiabetic drugs. One possible explanation is the inclusion of a more motivated nonuser cohort than would otherwise be expected in a general population: previous studies have suggested that in people with T2DM, the frequency of HbA_1c_ testing is low compared with guideline recommendations [17,18].

The improved glycemic control associated with DDS use occurred in addition to usual care, as an incremental benefit to patients, with no requirement to change drug therapy. The DDS has the potential to provide an additional tool for patients to improve their HbA_1c_ while undergoing standard care. Although it was not evaluated as part of this study, use of the DDS makes it possible for health care providers to monitor patients’ health and care episodes between appointments through reports that can be generated from the app [9], potentially informing the patient-physician consultation.

In this study, the DDS appears to have encouraged continuous patient engagement, with associated clinical improvement. Each day of patient engagement with any of the 10 meaningful activities in the DDS was associated with a greater drop in HbA_1c_. The study also indicated that use of the DDS increased adherence with medication, defined as the PDC. Baseline rates of adherence were already close to 80%, which is considered good adherence, but increased significantly more in DDS users than in nonusers. More analysis of subgroups is needed to further characterize the source of this improvement in medication adherence.

In diabetes care, any improvement in glycemic control with a new measure must be weighed against the intervention’s safety profile, specifically with respect to the occurrence of hypoglycemia. The study showed that there was no increase in the rates of severe hypoglycemia, defined as events requiring medical intervention and as recorded in claims data, among DDS users relative to baseline or to nonusers. The definition used did not provide a full picture of all hypoglycemia occurrences but captured the most severe events, which are the greatest concern to patients and have the greatest impact on resource use and costs to the health care system. Although mild hypoglycemia can be a substantial burden on patients with T2DM, it is often underreported in claims data, as patients may not seek out medical care or the hypoglycemic event may not be inputted to the claims database [19,20]. Therefore, including mild hypoglycemic events within the analysis may have reduced the accuracy of the results, but the impact of those events on patient outcomes should not considered inconsequential.

### Comparison With Prior Work

Several previous studies had shown that use of the DDS we studied is associated with improved BG control. For example, Hershcovitz et al [21] reported a single-arm retrospective study on use of the DDS in individuals with T2DM and with estimated HbA_1c_ (based on BG readings) >8% (n=148) or >9% (n=220). In that study, reduction≥1.5% in estimated HbA_1c_ versus baseline were reported and were sustained over 2 years, associated with a 20% reduction in glycemic variability. Similarly, a retrospective real-world single-arm analysis followed DDS users with average BG >180 mg/dL over 12 months and found that the average BG level decreased significantly over a year versus baseline, with no differences between White adults and those from racial and ethnic minority groups [22]. The results also showed a positive association between participants’ digital engagement and reduction in their glycemia. A retrospective real-world analysis of 998 DDS users found that monthly average BG levels improved significantly in the first 6 months of use, and this improvement was maintained during the following 6 months [23]. Moreover, the initial decrease in glycemia was steeper in individuals who frequently used other DDS features, in addition to BG measurement, compared with those who did not.

Randomized controlled trials of other DDS technologies have been conducted. For example, the efficacy and safety of a digital app delivering cognitive behavioral therapy was assessed in 326 adults with T2DM randomly assigned to use of the app and 343 control patients. Relative to the control arm, the app users had significantly greater reduction in HbA_1c_ from baseline at 90 days [24]. In another randomized controlled trial of an integrated digital health care platform with artificial intelligence–based dietary management, conducted over 48 weeks in South Korea with the participation of 294 adults with T2DM who were each assigned to one of three arms (routine diabetes care only, DDS alone, or DDS with feedback from medical staff and intermittently applied personal continuous glucose monitoring), use of that DDS was associated with better glycemic control and more weight loss [25]. However, such studies remain rare likely, in large part, because of the substantial resources and organization required to conduct them. Like previous studies of the Dario DDS, most assessments of other DDS technologies have used single-arm studies, such as that of a digitally enhanced diabetes self-management, education, and support program reported by Wilson-Anumudu et al [26], or may rely on the level of retention to stratify patients [27].

### Strengths and Limitations

This study had many strengths. Unlike the earlier retrospective studies of the DDS [21-23], this study is the first to include a comparator arm of DDS nonusers. The lack of a comparator arm has been a shortcoming in some studies on digital interventions, such as those analyzed by Kumar et al [8]. Furthermore, the study used well-matched cohorts more commonly associated with randomized controlled trials. This was achieved by linking data sources (including point-of-care data and patient-generated health data to provide a broad perspective) using anonymized and tokenized data, as well as the use of both exact matching and PSM to identify the DDS nonuser cohort, as described in detail elsewhere [10]. The matching methodology was designed to minimize any potential biases, with criteria (eg, comorbidities) chosen considering T2DM characteristics and outcomes. Hypertension and hyperlipidemia are risk factors for CVD and are additional proxies for disease severity/level of CVD risk, anemia affects blood HbA_1c_ levels, and depression is more prevalent among people with T2DM than those without and may be associated with HbA_1c_ levels. The comedications included in the PSM criteria reflected their use in the treatment of patients with these comorbidities [28,29].

As the study period overlapped with the COVID-19 pandemic, it was necessary to ensure that any impact on access to care did not affect the two cohorts differently. Potential biases were overcome by matching the two cohorts by quarter of the study period in which patients were enrolled. The study had a clinical primary endpoint, recognized as a factor that denotes rigor [8], and assessment of glycemic control relied on laboratory-reported HbA_1c_ rather than self-reported fasting BG. A 60-day window was allowed for HbA_1c_ tests to be conducted, which mirrors real-world practice.

The wide patient parameters used mean that the results may be generalizable, for example, to both commercially insured and Medicare-insured patients, as both were represented. Any adult with uncontrolled T2DM in the general US population who may be likely to use the DDS, which requires use of a smartphone, could potentially expect improved glycemic control. However, the study was biased toward more motivated patients in both cohorts, due to the need for baseline and follow-up HbA_1c_ measurements. Consequently, the effects associated with DDS use could potentially be even greater when compared with a less motivated population than the nonuser cohort of the study.

The study also has some limitations. First, as it was an observational study, residual confounding may be present. Baseline behavioral traits that contributed to glycemic control may have differed between DDS users and nonusers. An attempt to control for this by analyzing the use of test strips was limited because it was not possible to account for test strips that were self-paid or sent directly to users. Availability of baseline and follow-up HbA_1c_ measurements was an inclusion criterion, meaning that selection bias, for surviving patients who regularly have their data captured in interactions with health care services, is possible. There is the potential for comorbidities outside those included in the PSM criteria to be confounding, but there is a limit to the criteria that can be feasibly included in PSM, and there was a sound rationale to support the inclusion of the chosen comorbidities that do not apply to other comorbidities. Second, as in any study that relies on database entries, there may have been gaps or errors in the data. Although the coverage of the IDV is extensive (286 million patients in the United States), it is not universal, and as an open claims database, it may not have captured all health care events associated with enrolled patients. Third, individuals must be able to acquire the DDS, through employer benefits, health plan coverage, or the ability to self-pay, which could mean there are socioeconomic differences between DDS users and nonusers. Payer type was included in the PSM criteria, which should partially mitigate the socioeconomic difference between the two groups. Fourth, it was impossible to exclude patients using other digital coaching programs for diabetes, or other mobile technologies, from the DDS nonuser cohort where usage is not captured in clinical records or claims data. Capturing this information would require substantial changes to the IDV and is likely not feasible owing to the intended generalizability of the database. Finally, some subgroups served primarily to highlight directionality for future research and were not powered to show statistical significance. Nonsignificance of results in these subgroups, and between the two cohorts in laboratory values and some comprehensive diabetes health care measures, may have been due to the small sample size of subgroups. Expanding the size of the cohort population could increase the subgroup sample size, but in some cases, the size of population is inherent to the subgroup characteristics.

### Conclusion

Health outcomes are determined not only by medical care but also by behavior, social determinants of health, and genetic factors. Digital health interventions show promise in supporting desirable behavior in the self-management of diabetes [4-6] but have often lacked adequate evidence for their effectiveness [7]. This retrospective cohort study provided a rigorous assessment of the impact of a digital health solution on holistic patient management. It compared clinical outcomes of adults with T2DM using a digital health intervention, the Dario DDS, in addition to usual care, with those receiving usual care alone. The use of exact-matching and PSM minimized differences between the DDS user and nonuser cohorts.

At 6-month follow-up, improvements in glycemic control were statistically significantly greater among DDS users compared with nonusers, with no increase in the rate of severe hypoglycemia. The differences in glycemic control were maintained at 12 months, even though the study had not been powered to show this. Adherence with medication increased significantly more among DDS users than among nonusers at 6 months. At 12 months, the number of patients with good glycemic control increased and those with poor glycemic control decreased.

Future research and subgroup analyses of this patient population could identify patients who would most benefit from intervention with a DDS. As increased engagement correlated with change in HbA_1c_, efforts to identify ways to improve patient engagement with a DDS could further improve outcomes. Expanding the analysis to include patients outside the United States would increase the worldwide generalizability of the study, especially in relation to the differences in health care systems, lifestyle, and disease management.

The study provides evidence on the positive impact of a digital health intervention on the management of T2DM, with findings that are likely generalizable to a wide range of adults with T2DM. The results indicate that in a large cohort of patients with T2DM, digital health interventions that facilitate behavior change can deliver additional improvements to health outcomes, over and above the benefits conferred by drugs and disease education, that is not individually targeted.

## Appendix

Multimedia Appendix 1 Dario DDS: example displays. DDS: digital diabetes solution.

Multimedia Appendix 2 Methods (supplementary information).

Multimedia Appendix 3 Standardized mean differences in baseline characteristics of unmatched and matched individuals.

Multimedia Appendix 4 Sensitivity analysis: mean change in HbA_1c_ from baseline to 12-month follow-up in all patients and in subgroups by baseline HbA_1c_. HbA_1c_: glycated hemoglobin.

Multimedia Appendix 5 Percentage of patients achieving specific targets and mean change in HbA_1c_ from baseline to 6- and 12-month follow-up in selected subgroups. HbA_1c_: glycated hemoglobin.

Multimedia Appendix 6 Estimated HbA_1c_ reduction according to medication class. HbA_1c_: glycated hemoglobin.

Multimedia Appendix 7 Change in HbA_1c_ from baseline to the 6-month follow-up using individual engagement measures. HbA_1c_: glycated hemoglobin.

Multimedia Appendix 8 Change in diabetic comprehensive care measures at 12-month follow-up.

Multimedia Appendix 9 Change in laboratory values from baseline.

Multimedia Appendix 10 Change in CVD risk score. CVD: cardiovascular disease.

## Abbreviations

BG: blood glucose
BP: blood pressure
CVD: cardiovascular disease
DBP: diastolic blood pressure
DDS: digital diabetes solution
D-i-D: difference-in-difference
eGFR: estimated glomerular filtration rate
EMR: electronic medical records
GLM: generalized linear model
HbA_1c_: glycated hemoglobin
HEDIS: Healthcare Effectiveness Data and Information Set
IDV: Integrated Dataverse
LDL-c: low-density lipoprotein cholesterol
OAD: oral antidiabetic drug
PDC: proportion of days covered
PSM: propensity score matching
SBP: systolic blood pressure
T2DM: type 2 diabetes mellitus
VIF: variance inflation factor

## Data Availability

Qualified researchers may request access to data. Further details on Sanofi’s data-sharing criteria, eligible studies, and process for requesting access can be found online [30].

## References

[1] Karam SL, Dendy J, Polu S, Blonde L (2020). Overview of therapeutic inertia in diabetes: prevalence, causes, and consequences. Diabetes Spectr.

[2] Okemah J, Peng J, Quiñones M (2018). Addressing clinical inertia in type 2 diabetes mellitus: a review. Adv Ther.

[3] American Diabetes Association Professional Practice Committee (2025). Erratum. 5. Facilitating positive health behaviors and well-being to improve health outcomes: standards of care in diabetes-2025. Diabetes Care 2025; 48(Suppl. 1):S86-S127. Diabetes Care.

[4] Nkhoma DE, Soko CJ, Bowrin P, Manga YB, Greenfield D, Househ M, Li Jack Y, Iqbal U (2021). Digital interventions self-management education for type 1 and 2 diabetes: a systematic review and meta-analysis. Comput Methods Programs Biomed.

[5] Shan R, Sarkar S, Martin SS (2019). Digital health technology and mobile devices for the management of diabetes mellitus: state of the art. Diabetologia.

[6] Kerr D, King F, Klonoff DC (2019). Digital health interventions for diabetes: everything to gain and nothing to lose. Diabetes Spectr.

[7] Silberman J, Wicks P, Patel S, Sarlati S, Park S, Korolev IO, Carl JR, Owusu JT, Mishra V, Kaur M, Willey VJ, Sucala ML, Campellone TR, Geoghegan C, Rodriguez-Chavez IR, Vandendriessche B, Goldsack JC, Evidence DEFINED Workgroup (2023). Rigorous and rapid evidence assessment in digital health with the evidence DEFINED framework. NPJ Digit Med.

[8] Kumar A, Ross JS, Patel NA, Rathi V, Redberg RF, Dhruva SS (2023). Studies of prescription digital therapeutics often lack rigor and inclusivity. Health Aff (Millwood).

[9] (2023). Dario® blood glucose monitoring system. Digital Therapeutics Alliance.

[10] Thingalaya N, Kerr D, Potukuchi P, Wilson L, Lee K, Han-Burgess E, Edwards A, Yu X, Kennedy A, Lee F (2023). 962-P: impact of digital diabetes solution on glycemic control in adults with type 2 diabetes mellitus in the United States—a retrospective cohort study. Diabetes.

[11] (2023). Analyse and integrate data. ICON.

[12] (2023). What we do. Quest Diagnostics.

[13] ElSayed NA, Aleppo G, Aroda VR, Bannuru RR, Brown FM, Bruemmer D, Collins BS, Hilliard ME, Isaacs D, Johnson EL, Kahan S, Khunti K, Leon J, Lyons SK, Perry ML, Prahalad P, Pratley RE, Seley JJ, Stanton RC, Gabbay RA, on behalf of the American Diabetes Association (2023). 6. Glycemic targets: standards of care in diabetes-2023. Diabetes Care.

[14] (2023). 2023 diabetes recognition program requirements package. Healthcare Effectiveness Data and Information Set (HEDIS).

[15] D'Agostino RB, Vasan RS, Pencina MJ, Wolf PA, Cobain M, Massaro JM, Kannel WB (2008). General cardiovascular risk profile for use in primary care: the Framingham Heart Study. Circulation.

[16] (2025). Privacy policy. Dario Health.

[17] Duff CJ, Solis-Trapala I, Driskell OJ, Holland D, Wright H, Waldron JL, Ford C, Scargill JJ, Tran M, Hanna FWF, Pemberton RJ, Heald A, Fryer AA (2018). The frequency of testing for glycated haemoglobin, HbA_1c_, is linked to the probability of achieving target levels in patients with suboptimally controlled diabetes mellitus. Clin Chem Lab Med.

[18] Imai C, Li L, Hardie R, Georgiou A (2021). Adherence to guideline-recommended HbA_1c_ testing frequency and better outcomes in patients with type 2 diabetes: a 5-year retrospective cohort study in Australian general practice. BMJ Qual Saf.

[19] Liu J, Wang R, Ganz ML, Paprocki Y, Schneider D, Weatherall J (2018). The burden of severe hypoglycemia in type 2 diabetes. Curr Med Res Opin.

[20] Uzoigwe C, Hamersky CM, Arbit DI, Weng W, Radin MS (2020). Assessing prevalence of hypoglycemia in a medical transcription database. Diabetes Metab Syndr Obes.

[21] Hershcovitz Y, Dar S, Feniger E (2020). 895-P: estimated A_1c_ reduction in high-risk patients over two years of using a digital diabetes management platform. Diabetes.

[22] Gershoni T, Ritholz MD, Horwitz Dl, Manejwala O, Donaldson-Pitter T, Fundoiano-Hershcovitz Y (2022). Glycemic management by a digital therapeutic platform across racial/ethnic groups: a retrospective cohort study. Appl Sci.

[23] Fundoiano-Hershcovitz Y, Hirsch A, Dar S, Feniger E, Goldstein P (2021). Role of digital engagement in diabetes care beyond measurement: retrospective cohort study. JMIR Diabetes.

[24] Hsia J, Guthrie NL, Lupinacci P, Gubbi A, Denham D, Berman MA, Bonaca MP (2022). Randomized, controlled trial of a digital behavioral therapeutic application to improve glycemic control in adults with type 2 diabetes. Diabetes Care.

[25] Lee Y, Kim G, Jun JE, Park H, Lee WJ, Hwang Y, Kim JH (2023). An integrated digital health care platform for diabetes management with AI-based dietary management: 48-week results from a randomized controlled trial. Diabetes Care.

[26] Wilson-Anumudu F, Quan R, Castro Sweet C, Cerrada C, Juusola J, Turken M, Bradner Jasik C (2021). Early insights from a digitally enhanced diabetes self-management education and support program: single-arm nonrandomized trial. JMIR Diabetes.

[27] Tu Y, Chang Y, Chiou H, Lai K (2021). The effects of continuous usage of a diabetes management app on glycemic control in real-world clinical practice: retrospective analysis. J Med Internet Res.

[28] Beran M, Muzambi R, Geraets A, Albertorio-Diaz JR, Adriaanse MC, Iversen MM, Kokoszka A, Nefs G, Nouwen A, Pouwer F, Huber JW, Schmitt A, Schram MT, European Depression in Diabetes (EDID) Research Consortium (2022). The bidirectional longitudinal association between depressive symptoms and HbA: a systematic review and meta-analysis. Diabet Med.

[29] Ali S, Stone MA, Peters JL, Davies MJ, Khunti K (2006). The prevalence of co-morbid depression in adults with type 2 diabetes: a systematic review and meta-analysis. Diabet Med.

[30] A global clinical research data sharing platform. Vivli.

