# Supplementary material for: Clinical Effects of a Digital Health Intervention for Adults With Type 2 Diabetes in the United States: Retrospective Cohort Study
Source: J Med Internet Res. 2026 Jun 9;28:e66911. doi: 10.2196/66911 (PMC13291732; doi:10.2196/66911)
Supplement: Multimedia Appendix 2 [file jmir_v28i1e66911_app2.docx]

**Clinical Effects of a Digital Health Application in Patients with Type 2 Diabetes in the United States: A Retrospective Cohort Study**

**Multimedia Appendix 2: Methods (supplementary information)**

**Sample size:**

Based on the results of a meta-analysis, an absolute difference in the 6-month follow-up HbA1c of 0.3% was anticipated. For a conservative estimate, we used an standard deviation (SD) of 2.1 for the sample size calculation, based on descriptive statistics of HbA1c from samples of digital diabetes solution (DDS) users who were linked to laboratory data. With a matching ratio of 1:3, we calculated that 513 individuals were needed in the DDS user cohort and 1539 matched DDS non-users to achieve 80% power with alpha=0.05. A sequential matching strategy ensured that at least one DDS non-user was included for every DDS user.

**Data management:**
The Synoma^®^ Patient Integration process compiles data from different providers into an integrated data source. Tokenization is the process of replacing sensitive data with unique identification symbols that retain all the essential information without compromising its security. The Synoma^®^ tokenization engine de-identifies and integrates healthcare data so that they are linked back to a unique patient identifier, the Synoma ID. The process is compliant with the HIPAA. Data from all the sources described above were de-identified and tokenized with a Synoma ID before use in the study.

**Matching of DDS non-users and with DDS users:**

Once the DDS non-users who met all specifications of the study cohort and their index dates were identified, the cohort was further refined to the subset of individuals who matched at least one DDS user, based on both exact-matching criteria and propensity score matching (PSM) to account for confounding. Exact-matching criteria were: quarter in the study period in which index date fell, sex, payer, and antidiabetic medication type(s) (oral antidiabetic drugs only, insulin only, any other). DDS users were matched at a maximum 1:3 ratio, in a sequential manner. Covariates for both exact and PSM were assessed in the baseline period of 12 months before the index date.

PSM represented the predicted probability of being assigned to the DDS cohort, and was defined as logit propensity score within a certain distance, as estimated from a logistic regression model to calculate the predicted probability of being a DDS user. The model included baseline HbA1c, race, age, geographic region, Charlson Comorbidity Index (CCI) score, and relevant comorbidities, as follows: hypertension, hyperlipidemia, anemia, and depression. Co-medications (concomitant medications that could impact on blood glucose levels or are used in the relevant comorbidities) were independent variables. DDS user = 1 and DDS non-user = 0 was the dependent variable. A greedy nearest-neighbor matching algorithm without replacement matched users with non-users based off of the logit of propensity score using a caliper equal to 0.2 of the standard deviation (SD) of the logit of the propensity score.

PSM without replacement was performed, for a targeted ratio of one DDS user to three DDS non-users. Two approaches to PSM were compared: the first method included exact-matching variables in the propensity score model as independent variables, whereas the second did not. Model fit was assessed for both methods. The first method was established as the better approach to propensity scoring based on the Akaike Information Criterion for model fit.

For some individuals with missing medical data during the baseline period, measurement of characteristics relying on diagnosis codes (comorbidities and CCI score) was not achievable. These individuals were matched amongst themselves and separately from those with baseline diagnosis data. For these individuals, the propensity score model did not include comorbidities or CCI score.

After matching, the balance of covariates between matched DDS user and non-user cohorts was assessed by calculating the standardized mean differences in baseline characteristics between the two cohorts, to confirm it did not exceed 0.1.

**General statistical methods:**

All analyses were conducted under the assumption of intention-to-treat.

Continuous variables were statistically compared between the DDS user and non-user cohorts by t tests if the assumption of normality held, or Wilcoxon Rank Sum test if a non-parametric test was needed based on review of histograms. For categorical variables, study cohorts were compared using chi-squared tests or Fisher’s exact test, whichever was appropriate given cell sizes and number of categories.

To calculate the primary endpoint (change in HbA1c), a multivariable linear regression with a fixed-effect model was generated to determine the association between DDS users and follow-up HbA1c compared with DDS non-users. Baseline HbA1c and follow-up HbA1c were both included in the inclusion criteria so that there would be no missing data for these variables. Any missing data for the endpoints sourced from EMR data under the exploratory objectives were quantified in terms of the number of unique individuals with missing data, and values were not imputed.

For endpoints reported as change from baseline to follow-up, a difference-in-difference method was used. Change was defined as follow-up HbA1c value minus baseline HbA1c value. The baseline HbA1c value was the most recent test result measured during the baseline period ([Index Date –180 days] to [Index Date +30 days]). An additional 30 days were allowed so that HbA1c tests done close to registration would qualify as baseline values (as HbA1c levels indicate glycemia over the preceding 90 days, these tests would still reflect the immediate past). The additional 30 days also allowed for any time lag between the test and its result. The follow up HbA1c value was the most recent test result measured during the follow-up period ([Index Date +31 days] to [Index Date +240 days]) with a minimum time between baseline and follow-up of 90 days.
