# Supplementary material for: Clinical Effects of a Digital Health Intervention for Adults With Type 2 Diabetes in the United States: Retrospective Cohort Study
Source: J Med Internet Res. 2026 Jun 9;28:e66911. doi: 10.2196/66911 (PMC13291732; doi:10.2196/66911)
Supplement: Multimedia Appendix 3 [file jmir_v28i1e66911_app3.docx]

**Clinical Effects of a Digital Health Application in Patients with Type 2 Diabetes in the United States: A Retrospective Cohort Study**

**Multimedia Appendix 3**

**Table S1. Standardized mean differences in baseline characteristics of unmatched and matched individuals.**

|  | **Unmatched** | | | | | | **Matched** | | | |
| --- | --- | --- | --- | --- | --- | --- | --- | --- | --- | --- |
|  | **DDS Users and Non-Users with Baseline Diagnosis Data** | | | **DDS Users and Non-Users with No Baseline Diagnosis Data** | | | **DDS Users and Non-Users with Baseline Diagnosis Data** | | **DDS Users and Non-Users with No Baseline Diagnosis Data** | |
| **Variable** | **Mean Difference^a^** | **SD** | **Standardized Difference^b^** | **Mean Difference^a^** | **SD** | **Standardized Difference^b^** | **Mean Difference^a^** | **Standardized Difference^b^** | **Mean Difference^a^** | **Standardized Difference^b^** |
|  |  |  |  |  |  |  |  |  |  |  |
| Logit Propensity Score | 1.04195 | 1.29489 | 0.80466 | 0.66695 | 1.14687 | 0.58154 | 0.0019 | 0.00146 | -0.00044 | -0.00039 |
| Baseline HbA1c | 0.68329 | 1.61228 | 0.42381 | 0.68329 | 1.61228 | 0.42381 | 0.00236 | 0.00146 | 0.01539 | 0.00955 |
| Age | -6.13643 | 11.31954 | -0.54211 | -6.13643 | 11.31954 | -0.54211 | -0.32631 | -0.02883 | 0.48643 | 0.04297 |
| Sex | -0.01463 | 0.49925 | -0.02931 | -0.01463 | 0.49925 | -0.02931 | -0.00036 | -0.00073 | 0.0001 | 0.0002 |
| Anemia | 0.01187 | 0.20805 | 0.05706 | 0.01187 | 0.20805 | 0.05706 | -0.00928 | -0.0446 | -0.00299 | -0.01436 |
| Hypertension | 0.02351 | 0.49412 | 0.04758 | 0.02351 | 0.49412 | 0.04758 | 0.03302 | 0.06683 | -0.00524 | -0.01061 |
| Hyperlipidemia | 0.01774 | 0.49992 | 0.03549 | 0.01774 | 0.49992 | 0.03549 | 0.0128 | 0.02559 | -0.01635 | -0.03271 |
| Depression | 0.00118 | 0.26313 | 0.0045 | 0.00118 | 0.26313 | 0.0045 | -0.00227 | -0.00863 | -0.00055 | -0.00208 |
| Anxiolytics | -0.11456 | 0.45865 | -0.24978 | -0.11456 | 0.45865 | -0.24978 | 0.00028 | 0.00062 | -0.02453 | -0.05347 |
| Anemia medications | 0.00916 | 0.11683 | 0.07842 | 0.00916 | 0.11683 | 0.07842 | -0.0062 | -0.05306 | -0.00541 | -0.04635 |
| Hypertension medications | 0.01724 | 0.39109 | 0.04408 | 0.01724 | 0.39109 | 0.04408 | 0.027 | 0.06904 | 0.00172 | 0.00439 |
| Steroids | -0.01992 | 0.43417 | -0.04588 | -0.01992 | 0.43417 | -0.04588 | -0.01143 | -0.02633 | -0.00618 | -0.01424 |
| Dyslipidemia medications | 0.02389 | 0.44556 | 0.05361 | 0.02389 | 0.44556 | 0.05361 | -0.00565 | -0.01268 | -0.01799 | -0.04038 |
| Proton Pump Inhibitors | -0.0003 | 0.43458 | -0.00073 | -0.0003 | 0.4346 | -0.00073 | -0.0037 | -0.00847 | -0.007 | -0.01608 |

DDS: Digital Diabetes Solution; SD: standard deviation; HbA1c: hemoglobin A1c

^a^ Mean Differences = DDS User - DDS Non-Users

^b^ Standard deviation of Unmatched observations used to compute standardized differences
