# Supplementary material for: Clinical Effects of a Digital Health Intervention for Adults With Type 2 Diabetes in the United States: Retrospective Cohort Study
Source: J Med Internet Res. 2026 Jun 9;28:e66911. doi: 10.2196/66911 (PMC13291732; doi:10.2196/66911)
Supplement: Multimedia Appendix 4 [file jmir_v28i1e66911_app4.docx]

**Clinical Effects of a Digital Health Application in Patients with Type 2 Diabetes in the United States: A Retrospective Cohort Study**

**Multimedia Appendix 4**

**Table S1. Sensitivity analysis: mean change in HbA1c from baseline to 12-month follow-up, in all patients and in subgroups by baseline HbA1c.**

|  |  |  |  | **Baseline** | | **Follow-up** | | **Difference-in-difference** | ***P* value** |
| --- | --- | --- | --- | --- | --- | --- | --- | --- | --- |
|  |  |  |  | **DDS users** | **DDS non-users** | **DDS users** | **DDS non-users** |  |  |
|  |  |  |  |  |  |  |  |  |  |
| **Sensitivity analysis: All** | | | | | | | | | |
|  | N | | | 345 | 1070 | 345 | 1070 |  |  |
|  | HbA1c, mean (SD) | | | 8.96 (1.74) | 8.95 (1.77) | 8.00 (1.64) | 8.22 (1.74) |  |  |
|  |  | Change from baseline (GLM) | | | | | | | |
|  |  |  | Mean |  |  | −0.96 | −0.74 | −0.22 | **.03** |
|  |  |  | 95% CI |  |  | (−1.12, −0.79) | (−0.83, −0.64) | (−0.41, −0.03) |  |
|  |  |  | *P* value |  |  | <.0001 | <.0001 |  |  |
| **Sensitivity analysis of subgroup: HbA1c >7.5% at baseline** | | | | | | | | | |
|  | N | | | 270 | 809 | 270 | 809 |  |  |
|  | HbA1c, mean (SD) | | | 9.43 (1.69) | 9.50 (1.70) | 8.19 (1.68) | 8.48 (1.83) |  |  |
|  |  | Change from baseline (GLM) | | | | | | | |
|  |  |  | Mean |  |  | −1.28 | −1.01 | −0.26 | **.03** |
|  |  |  | 95% CI |  |  | (−1.48, −1.07) | (−1.13,−0.89) | (−0.50, −0.03) |  |
|  |  |  | *P* value |  |  | <.0001 | <.0001 |  |  |
| **Sensitivity analysis of subgroup: HbA1c >8% at baseline** | | | | | | | | | |
|  | N | | | 215 | 624 | 215 | 624 |  |  |
|  | HbA1c, mean (SD) | | | 9.85 (1.65) | 10.01 (1.61) | 8.36 (1.74) | 8.71 (1.90) |  |  |
|  |  | Change from baseline (GLM) | | | | | | | |
|  |  |  | Mean |  |  | −1.58 | −1.28 | −0.30 | **.03** |
|  |  |  | 95% CI |  |  | (−1.82, −1.34) | (−1.42, −1.13) | (−0.58, −0.02) |  |
|  |  |  | *P* value |  |  | <.0001 | <.0001 |  |  |
| **Sensitivity analysis of subgroup: HbA1c >9% at baseline** | | | | | | | | | |
|  | N | | | 121 | 400 | 121 | 400 |  |  |
|  | HbA1c, mean (SD) | | | 10.88 (1.52) | 10.85 (1.43) | 8.50 (1.90) | 9.08 (2.05) |  |  |
|  |  | Change from baseline (GLM) | | | | | | | |
|  |  |  | Mean |  |  | −2.37 | −1.78 | −0.59 | **.0047** |
|  |  |  | 95% CI |  |  | (−2.72, −2.01) | (−1.97, −1.58) | (−0.99, −0.18) |  |
|  |  |  | P value |  |  | <.0001 | <.0001 |  |  |
| **Sensitivity analysis of subgroup: HbA1c >11% at baseline** | | | | | | | | | |
|  | N | | | 48 | 150 | 48 | 150 |  |  |
|  | HbA1c, mean (SD) | | | 12.52 (0.91) | 12.42 (1.00) | 8.65 (2.32) | 9.46 (2.20) |  |  |
|  |  | Change from baseline (GLM) | | | | | | | |
|  |  |  | Mean |  |  | −3.80 | −2.98 | −0.82 | **.03** |
|  |  |  | 95% CI |  |  | (−4.44, −3.16) | (−3.34, −2.62) | (−1.55, −0.09) |  |
|  |  |  | *P* value |  |  |  |  |  |  |

DDS: digital diabetes solution; GLM: generalized linear model.
