# Supplementary material for: Clinical Effects of a Digital Health Intervention for Adults With Type 2 Diabetes in the United States: Retrospective Cohort Study
Source: J Med Internet Res. 2026 Jun 9;28:e66911. doi: 10.2196/66911 (PMC13291732; doi:10.2196/66911)

**Clinical Effects of a Digital Health Application in Patients with Type 2 Diabetes in the United States: A Retrospective Cohort Study**

**Multimedia Appendix 5**

**Table S1. Percentage of patients achieving specific targets and mean change in HbA1c from baseline to 6- and 12-month follow-up in selected subgroups.**

|  |  |  |  | **Baseline** | | **Follow-up** | | **Difference-in-difference** | ***P* value** |
| --- | --- | --- | --- | --- | --- | --- | --- | --- | --- |
|  |  |  |  | **DDS users** | **DDS non-users** | **DDS users** | **DDS non-users** |  |  |
|  |  |  |  |  |  |  |  |  |  |
| **Patients with ≥1% decrease in HbA1c from baseline, 6 months** | | | | | | | | | |
|  | N | | | 267 | 625 | 267 | 625 |  |  |
|  | % of total patients | | | 47.0 | 36.8 | 47.0 | 36.8 | 10.2 | **<.001** |
|  | HbA1c, mean (SD) | | | 10.04 (1.90) | 10.26 (1.90) | 7.42 (1.56) | 7.55 (1.45) |  |  |
| **Patients with ≥1% decrease in HbA1c from baseline, 12 months** | | | | | | | | | |
|  | N | | | 151 | 413 | 151 | 413 |  |  |
|  | % of total patients | | | 26.6 | 24.3 | 26.6 | 24.3 |  | .12 |
|  | HbA1c, mean (SD) | | | 9.81 (1.93) | 9.97 (1.94) | 7.17 (1.3) | 7.42 (1.48) |  |  |
| **Patients on antidiabetic drugs other than insulin, 6 months** | | | | | | | | | |
|  | N | | | 349 | 1069 | 349 | 1069 |  |  |
|  | HbA1c, mean (SD) | | | 8.98 (1.75) | 8.93 (1.83) | 7.82 (1.59) | 8.12 (1.69) |  |  |
|  |  | Change from baseline (GLM) | | |  |  |  |  |  |
|  |  |  | Mean |  |  | −1.13 | −0.82 | −0.31 | **.001** |
|  |  |  | 95% CI |  |  | (−1.30, −0.97) | (−0.91, −0.72) | (−0.50, −0.13) |  |
| **Patients on antidiabetic drugs other than insulin, 12 months** | | | | | | | | | |
|  | N | | | 209 | 661 | 209 | 661 |  |  |
|  | HbA1c, mean (SD) | | | 8.81 (1.74) | 8.73 (1.73) | 7.81 (1.58) | 7.94 (1.64) |  |  |
|  |  | Change from baseline (GLM) | | |  |  |  |  |  |
|  |  |  | Mean |  |  | −0.96 | −0.80 | −0.16 | .19 |
|  |  |  | 95% CI |  |  | (−1.17, −0.75) | (−0.92, −0.68) | (−0.40, 0.08) |  |
|  |  |  |  |  |  |  |  |  |  |
| **Patients using test strips, 6 months** | | | | | | | | | |
|  | N | | | 172 | 633 | 172 | 633 |  |  |
|  | HbA1c, mean (SD) | | | 8.99 (1.76) | 9.05 (1.82) | 8.13 (1.71) | 8.22 (1.71) |  |  |
|  |  | Change from baseline (GLM) | | |  |  |  |  |  |
|  |  |  | Mean |  |  | −0.89 | −0.82 | −0.07 | .62 |
|  |  |  | 95% CI |  |  | (−1.13, −0.65) | (−0.95, −0.70) | (−0.34, 0.20) |  |
| **Patients using test strips, 12 months** | | | | | | | | | |
|  | N | | | 116 | 405 | 116 | 405 |  |  |
|  | HbA1c, mean (SD) | | | 8.78 (1.66 | 8.89 (1.71 | 8.03 (1.57 | 8.11 (1.65 |  |  |
|  |  | Change from baseline (GLM) | | |  |  |  |  |  |
|  |  |  | Mean |  |  | −0.80 | −0.77 | −0.04 | .83 |
|  |  |  | 95% CI |  |  | (−1.08, −0.53) | (−0.91, −0.62) | (−0.35, 0.28) |  |
| **Patients with severe hypoglycemia who reached HbA1c target of <8.0%, 12 months** | | | | | | | | | |
|  | Patients with baseline HbA1c ≥8%, n | | | 2 | 5 | 2 | 5 |  |  |
|  | Patients reaching HbA1c <8%, n | | | – | – | 1 | 3 |  |  |
|  | % reaching HbA1c <8% | | | – | – | 50.0 | 60.0 |  | .81 |
| **Patients without severe hypoglycemia who reached HbA1c target of <8.0%, 12 months** | | | | | | | | | |
|  | Patients with baseline HbA1c ≥8%, n | | | 225 | 648 | 225 | 648 |  |  |
|  | Patients reaching HbA1c <8%, n | | | – | – | 107 | 264 |  |  |
|  | % reaching HbA1c <8% | | | – | – | 47.6 | 40.7 |  | .07 |

DDS: digital diabetes solution; GLM: generalized linear model.

**Table S2.** Estimated HbA1c reduction according to medication class.

| **Medication class** | **Sample Size**  **Users vs non-users** | **Difference in difference**  **DDS users vs non-users**  **Estimate [CI] (*P*-value)** |
| --- | --- | --- |
|  |  |  |
| Overall model (controlling for baseline medication class) | 498 vs 1494 | −0.28 [−0.48, −0.08] (*P*=**.01**) |
| OAD only | 241 vs 749 | −0.27 [−0.56, 0.02] (*P*=.07) |
| Insulin only | 35 vs 106 | 0.15 [−0.52, 0.82] (*P*=.65) |
| Other | 3 vs 15 | −2.93 [−6.39, 0.54] (*P*=.09) |
| Any combination (OAD, insulin, and other injectables) | 219 vs 624 | −0.32 [−0.62, −0.02] (*P*=**.04**) |

DDS: digital diabetes solution; OAD: oral antidiabetic drug.

**Table S3.** Change in HbA1c from Baseline to the 6-Month Follow-up Using Individual Engagement Measures

|  | β-coefficient | Lower 95% CI | Upper 95% CI | *P*-value |
| --- | --- | --- | --- | --- |
|  | | | | |
| Baseline HbA1c | –0.706 | –0.775 | –0.636 | **<.001** |
| Measuring BG | –0.009 | –0.012 | –0.006 | **<.001** |
| Measuring BP | –0.002 | –0.009 | 0.005 | .59 |
| Measuring weight | 0.002 | –0.007 | 0.011 | .64 |
| Tagging (timing BG and meal type) | –0.005 | –0.008 | –0.001 | **.01** |
| Food logging (carb counting, meal photos, etc) | –0.006 | –0.016 | 0.004 | .20 |
| Inputting insulin dose | 0.005 | 0.000 | 0.010 | **.04** |
| Recording physical activity | –0.009 | –0.041 | 0.024 | .61 |
| Sharing logbook | 0.002 | –0.051 | 0.054 | .96 |
| Reading an article | 0.001 | –0.019 | 0.021 | .95 |
| Interacting with a coach | –0.062 | –0.249 | 0.126 | .52 |

BG, blood glucose; BP, blood pressure; CI, confidence interval.

**Table S4.** Change in diabetic comprehensive care measures at 12-month follow-up.

|  | Baseline | | Follow-up | | *P* value^a^ |
| --- | --- | --- | --- | --- | --- |
|  | DDS users | DDS non-users | DDS users | DDS non-users |  |
|  | | | | | |
| Patients with BL HbA1c ≥8.0% and follow-up HbA1c <8.0% | 387 | 1089 | 179 (46.3% of BL) | 419 (38.7% of BL) | **.01** |
| Patients with baseline and follow-up HbA1c >9.0% | 237 | 713 | 86 (36.3% of BL) | 347 (48.7% of BL) | **<.001** |
| All patients with BP at baseline; controlled BP (<140/99 mmHg) at follow-up | 120 | 346 | 93 (77.5% of BL) | 250 (72.3% of BL) | .26 |
| Patients with uncontrolled BP (≥140 mmHg and/or ≥90 mmHg) at baseline and controlled BP (<140/90 mmHg) at follow-up | 32 | 99 | 21 (65.6% of BL) | 49 (49.5% of BL) | .11 |
| Patients with nephropathy monitoring | 4 (0.7% of total) | 23 (1.4% of of total) | 9 (1.6% of total) | 46 (2.7% of total) | Baseline: .22 Follow-up: .13 |
| Patients with retinopathy exams | 3 (0.5% of total) | 9 (0.5% of total) | 3 (0.5% of total) | 4 (0.2% of total) | Baseline: 1.00 Follow-up: .28 |

^a^ *P*-value uses Chi-square test to compare the percentage difference between DDS users and non-users.

BL: baseline; BP: blood pressure; DDS: digital diabetes solution.

**Table S5**. Change in laboratory values from baseline.

|  |  |  |  | Baseline^a^ | | Follow-up^b^ | | Difference-in-difference | *P* value |
| --- | --- | --- | --- | --- | --- | --- | --- | --- | --- |
|  |  |  |  | DDS users | DDS non-users | DDS users | DDS non-users |  |  |
|  | | | | | | | | | |
| **EMR measurement – blood glucose** | | | | | | | | | |
|  | N | | | 78 | 193 | 78 | 193 |  |  |
|  | % of patients | | | 13.7 | 11.4 | 13.7 | 11.4 |  |  |
|  | Mean (mg/dL) | | | 201.99 | 200.36 | 169.65 | 178.83 |  |  |
|  | SD (mg/dL) | | | 72.94 | 94.73 | 69.65 | 87.97 |  |  |
|  | Change from baseline^c^ (mg/dL) | | |  |  |  |  | –10.81 | .11 |
| **EMR measurement – total cholesterol** | | | | | | | | | |
|  | N | | | 52 | 125 | 52 | 125 |  |  |
|  | % of patients | | | 9.2 | 7.4 | 9.2 | 7.4 |  |  |
|  | Mean (mg/dL) | | | 174.27 | 171.02 | 171.35 | 160.98 |  |  |
|  | SD (mg/dL) | | | 56.66 | 45.20 | 52.47 | 43.86 |  |  |
|  | Change from baseline^c^ (mg/dL) | | |  |  |  |  | 7.12 | .76 |
| **EMR measurement – triglycerides** | | | | | | | | | |
|  | N | | | 55 | 122 | 55 | 122 |  |  |
|  | % of patients | | | 9.7 | 7.2 | 9.7 | 7.2 |  |  |
|  | Mean (mg/dL) | | | 197.55 | 199.12 | 188.93 | 186.17 |  |  |
|  | SD (mg/dL) | | | 98.07 | 131.59 | 142.36 | 124.95 |  |  |
|  | Change from baseline^c^ (mg/dL) | | |  |  |  |  | 4.33 | .61 |
| **EMR measurement – LCL-c** | | | | | | | | | |
|  | N | | | 52 | 120 | 52 | 120 |  |  |
|  | % of patients | | | 9.2 | 7.1 | 9.2 | 7.1 |  |  |
|  | Mean (mg/dL) | | | 98.79 | 92.55 | 92.56 | 85.4 |  |  |
|  | SD (mg/dL) | | | 47.88 | 35.66 | 40.75 | 32.54 |  |  |
|  | Change from baseline^c^ (mg/dL) | | |  |  |  |  | 0.92 | .82 |
| **EMR measurement – eGFR** | | | | | | | | | |
|  | N | | | 57 | 148 | 57 | 148 |  |  |
|  | % of patients | | | 10.0 | 8.7 | 10.0 | 8.7 |  |  |
|  | Mean (mL/min/1.73m^2^) | | | 89.68 | 91.36 | 89.03 | 89.75 |  |  |
|  | SD (mL/min/1.73m^2^) | | | 27.31 | 30.34 | 27.53 | 29.81 |  |  |
|  | Change from baseline^c^ (mL/min/1.73m^2^) | | |  |  |  |  | 0.96 | .63 |
| **EMR measurement – SBP** | | | | | | | | | |
|  | N | | | 111 | 325 | 111 | 325 |  |  |
|  | % of patients | | | 19.5 | 19.1 | 19.5 | 19.1 |  |  |
|  | Mean (mmHg) | | | 130.09 | 130.86 | 128.47 | 131.40 |  |  |
|  | SD (mmHg) | | | 14.13 | 15.93 | 13.88 | 15.86 |  |  |
|  | Change from baseline^c^ (mmHg) | | |  |  |  |  | –2.16 | .45 |
| **EMR measurement – DBP** | | | | | | | | | |
|  | N | | | 111 | 325 | 111 | 325 |  |  |
|  | % of patients | | | 19.5 | 19.1 | 19.5 | 19.1 |  |  |
|  | Mean (mmHg) | | | 78.43 | 77.51 | 77.22 | 77.43 |  |  |
|  | SD (mmHg) | | | 10.12 | 9.63 | 10.47 | 10.34 |  |  |
|  | Change from baseline^c^ (mmHg) | | |  |  |  |  | –1.13 | .35 |
| **EMR measurement – average SBP** | | | | | | | | | |
|  | N | | | 111 | 325 | 111 | 325 |  |  |
|  | % of patients | | | 19.5 | 19.1 | 19.5 | 19.1 |  |  |
|  | Mean (mmHg) | | | 129.50 | 130.76 | 128.95 | 131.43 |  |  |
|  | SD (mmHg) | | | 11.28 | 12.75 | 11.08 | 13.96 |  |  |
|  | Change from baseline^c^ (mmHg) | | |  |  |  |  | –1.22 | .55 |
| **EMR measurement – average DBP** | | | | | | | | | |
|  | N | | | 111 | 325 | 111 | 325 |  |  |
|  | % of patients | | | 19.5 | 19.1 | 19.5 | 19.1 |  |  |
|  | Mean (mmHg) | | | 78.4 | 78.0 | 77.3 | 77.4 |  |  |
|  | SD (mmHg) | | | 8.25 | 7.98 | 8.31 | 8.70 |  |  |
|  | Change from baseline^c^ (mmHg) | | |  |  |  |  | –0.48 | .30 |
| **EMR measurement** – **weight** | | | | | | | | | |
|  | N | | | 112 | 327 | 112 | 327 |  |  |
|  | % of patients | | | 19.7 | 19.3 | 19.7 | 19.3 |  |  |
|  | Mean (lbs) | | | 224.89 | 219.27 | 223.18 | 219.17 |  |  |
|  | SD (lbs) | | | 47.39 | 50.03 | 47.04 | 48.93 |  |  |
|  | Change from baseline^c^ (lbs) | | |  |  |  |  | –1.61 | .80 |
| **EMR measurement – BMI** | | | | | | | | | |
|  | N | | | 111 | 318 | 111 | 318 |  |  |
|  | % of patients | | | 19.5 | 18.7 | 19.5 | 18.7 |  |  |
|  | Mean (kg/m^2^) | | | 34.64 | 34.11 | 34.43 | 34.14 |  |  |
|  | SD (kg/m^2^) | | | 6.16 | 6.38 | 6.19 | 6.35 |  |  |
|  | Change from baseline^c^ (kg/m^2^) | | |  |  |  |  | –0.24 | .92 |

^a^ Highest value on the last date.

^a^ Highest value on the date closest to the index date.

^c^ Difference-in-difference is defined as DDS user change minus non-user change. *P*-value uses nonparametric Wilcoxon rank test to compare change of measurement (follow-up minus baseline) for each patient between DDS users and non-users.

BP: blood pressure; DBP: diastolic blood pressure; DDS: digital diabetes solution; eGFR: estimated glomerular filtration rate; EMR: electronic medical records; LDL-c: low-density lipoprotein cholesterol; SBP: systolic blood pressure.

**Table S6.** Change in CVD risk score.

|  | | DDS users | DDS non-users | DDS users | DDS non-users | *P* value for baseline |
| --- | --- | --- | --- | --- | --- | --- |
|  | | | | | | |
| **Baseline** | | | | | | |
|  | Patients, n | 87 | 245 |  |  |  |
|  | Mean for Baseline: Index Date –365 days to Index Date +30 days |  |  | 16.49 | 16.98 | .32^a^ |
|  | Low FRS <10%, n (%) |  |  | 11 (12.6) | 30 (12.2) | .23^a^ |
|  | Intermediate FRS 10% to <20%, n (%) |  |  | 30 (34.5) | 62 (25.3) |  |
|  | High FRS ≥20%, n (%) |  |  | 46 (52.9) | 153 (62.5) |  |
| **12-month follow-up** | | | | | | |
|  | Patients, n | 87 | 250 |  |  |  |
|  | Mean for Baseline: Index Date –365 days to Index Date +30 days |  |  | 16.39 | 17.02 | .25^a^ |
|  | Low FRS <10%, n (%) |  |  | 13 (14.9) | 26 (10.4) | .52^a^ |
|  | Intermediate FRS 10% to <20%, n (%) |  |  | 12 (26.4) | 70 (28.0) |  |
|  | High FRS ≥20%, n (%) |  |  | 51 (59.6) | 154 (61.6) |  |

^a^ CVD score calculation method.

CVD: cardiovascular disease; DDS: digital diabetes solution; FRS: Framingham Risk Score.

Figure S1. Dario Diabetes Solution: Example displays


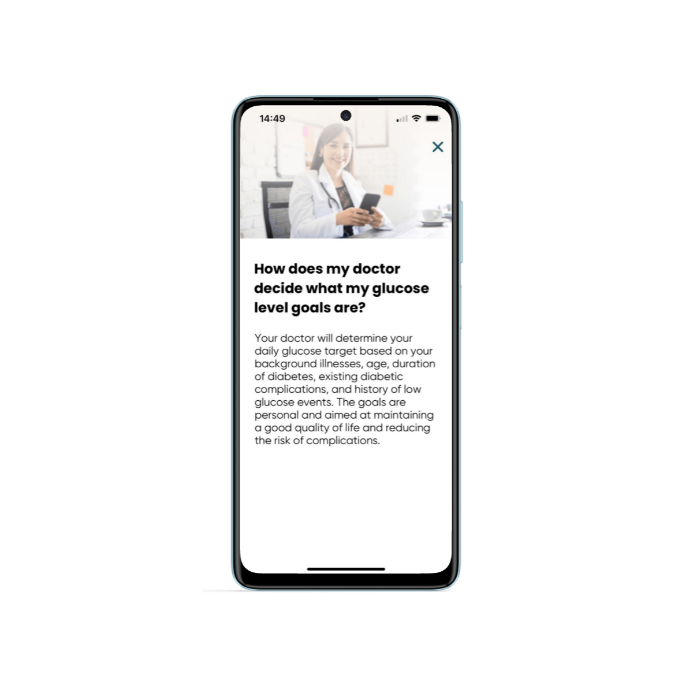

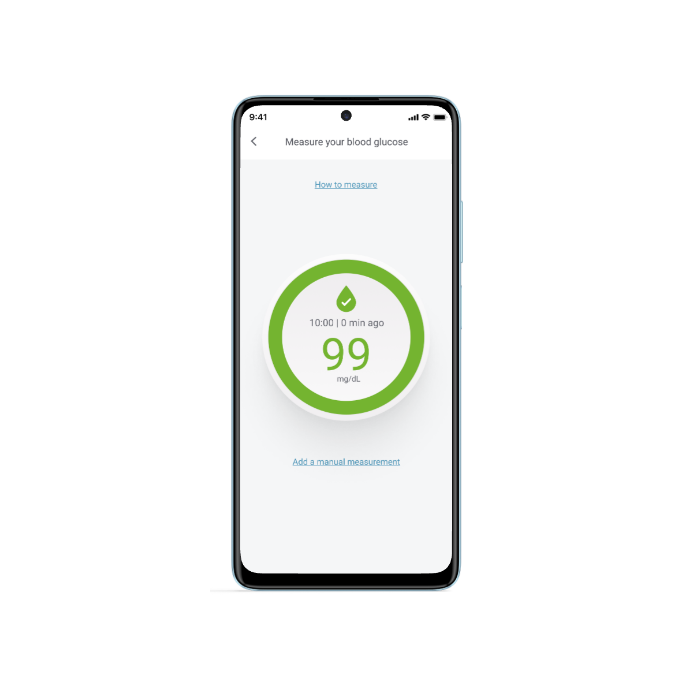

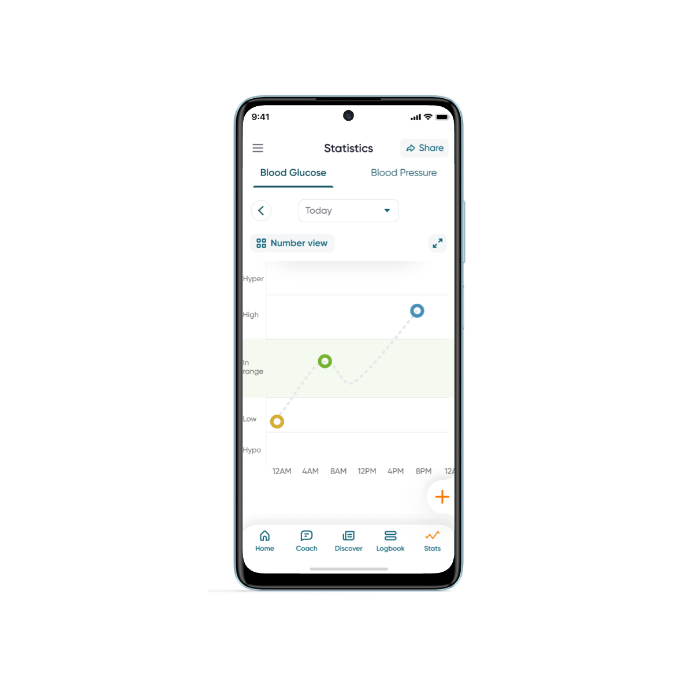

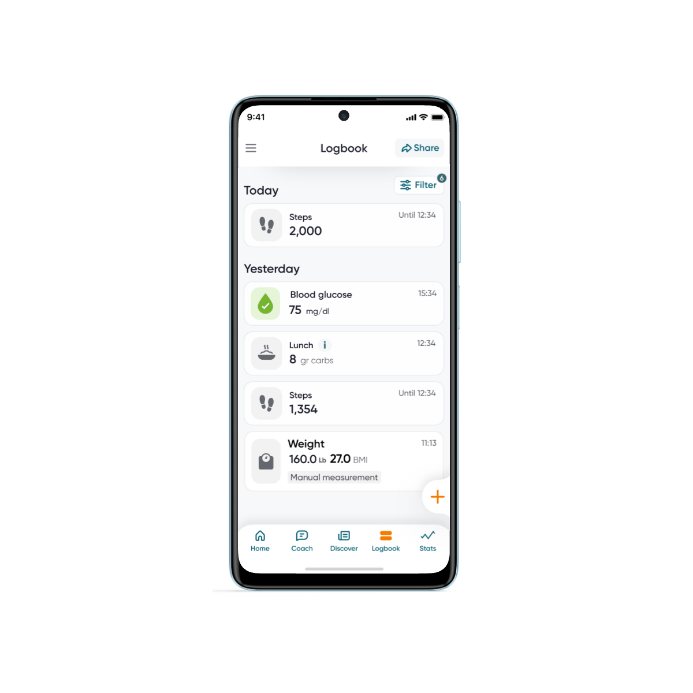

Supplement: Multimedia Appendix 5 [file jmir_v28i1e66911_app5.docx]
