# Supplementary material for: Clinical Effects of a Digital Health Intervention for Adults With Type 2 Diabetes in the United States: Retrospective Cohort Study
Source: J Med Internet Res. 2026 Jun 9;28:e66911. doi: 10.2196/66911 (PMC13291732; doi:10.2196/66911)
Supplement: Multimedia Appendix 6 [file jmir_v28i1e66911_app6.docx]

**Clinical Effects of a Digital Health Application in Patients with Type 2 Diabetes in the United States: A Retrospective Cohort Study**

**Multimedia Appendix 6**

**Table S1. Estimated HbA1c reduction according to medication class.**

| **Medication class** | **Sample Size**  **Users vs non-users** | **Difference in difference**  **DDS users vs non-users**  **Estimate [CI] (*P*-value)** |
| --- | --- | --- |
|  |  |  |
| Overall model (controlling for baseline medication class) | 498 vs 1494 | −0.28 [−0.48, −0.08] (*P*=**.01**) |
| OAD only | 241 vs 749 | −0.27 [−0.56, 0.02] (*P*=.07) |
| Insulin only | 35 vs 106 | 0.15 [−0.52, 0.82] (*P*=.65) |
| Other | 3 vs 15 | −2.93 [−6.39, 0.54] (*P*=.09) |
| Any combination (OAD, insulin, and other injectables) | 219 vs 624 | −0.32 [−0.62, −0.02] (*P*=**.04**) |

DDS: digital diabetes solution; OAD: oral antidiabetic drug.
