# Supplementary material for: Clinical Effects of a Digital Health Intervention for Adults With Type 2 Diabetes in the United States: Retrospective Cohort Study
Source: J Med Internet Res. 2026 Jun 9;28:e66911. doi: 10.2196/66911 (PMC13291732; doi:10.2196/66911)
Supplement: Multimedia Appendix 7 [file jmir_v28i1e66911_app7.docx]

**Clinical Effects of a Digital Health Application in Patients with Type 2 Diabetes in the United States: A Retrospective Cohort Study**

**Multimedia Appendix 7**

**Table S1. Change in HbA1c from baseline to the 6-month follow-up using individual engagement measures.**

|  | β-coefficient | Lower 95% CI | Upper 95% CI | *P*-value |
| --- | --- | --- | --- | --- |
|  | | | | |
| Baseline HbA1c | –0.706 | –0.775 | –0.636 | **<.001** |
| Measuring BG | –0.009 | –0.012 | –0.006 | **<.001** |
| Measuring BP | –0.002 | –0.009 | 0.005 | .59 |
| Measuring weight | 0.002 | –0.007 | 0.011 | .64 |
| Tagging (timing BG and meal type) | –0.005 | –0.008 | –0.001 | **.01** |
| Food logging (carb counting, meal photos, etc) | –0.006 | –0.016 | 0.004 | .20 |
| Inputting insulin dose | 0.005 | 0.000 | 0.010 | **.04** |
| Recording physical activity | –0.009 | –0.041 | 0.024 | .61 |
| Sharing logbook | 0.002 | –0.051 | 0.054 | .96 |
| Reading an article | 0.001 | –0.019 | 0.021 | .95 |
| Interacting with a coach | –0.062 | –0.249 | 0.126 | .52 |

BG, blood glucose; BP, blood pressure; CI, confidence interval.
