# Supplementary material for: Clinical Effects of a Digital Health Intervention for Adults With Type 2 Diabetes in the United States: Retrospective Cohort Study
Source: J Med Internet Res. 2026 Jun 9;28:e66911. doi: 10.2196/66911 (PMC13291732; doi:10.2196/66911)
Supplement: Multimedia Appendix 8 [file jmir_v28i1e66911_app8.docx]

**Clinical Effects of a Digital Health Application in Patients with Type 2 Diabetes in the United States: A Retrospective Cohort Study**

**Multimedia Appendix 8**

**Table S1. Change in diabetic comprehensive care measures at 12-month follow-up.**

|  | Baseline | | Follow-up | | *P* value^a^ |
| --- | --- | --- | --- | --- | --- |
|  | DDS users | DDS non-users | DDS users | DDS non-users |  |
|  | | | | | |
| Patients with BL HbA1c ≥8.0% and follow-up HbA1c <8.0% | 387 | 1089 | 179 (46.3% of BL) | 419 (38.7% of BL) | **.01** |
| Patients with baseline and follow-up HbA1c >9.0% | 237 | 713 | 86 (36.3% of BL) | 347 (48.7% of BL) | **<.001** |
| All patients with BP at baseline; controlled BP (<140/99 mmHg) at follow-up | 120 | 346 | 93 (77.5% of BL) | 250 (72.3% of BL) | .26 |
| Patients with uncontrolled BP (≥140 mmHg and/or ≥90 mmHg) at baseline and controlled BP (<140/90 mmHg) at follow-up | 32 | 99 | 21 (65.6% of BL) | 49 (49.5% of BL) | .11 |
| Patients with nephropathy monitoring | 4 (0.7% of total) | 23 (1.4% of of total) | 9 (1.6% of total) | 46 (2.7% of total) | Baseline: .22 Follow-up: .13 |
| Patients with retinopathy exams | 3 (0.5% of total) | 9 (0.5% of total) | 3 (0.5% of total) | 4 (0.2% of total) | Baseline: 1.00 Follow-up: .28 |

^a^ *P*-value uses Chi-square test to compare the percentage difference between DDS users and non-users.

BL: baseline; BP: blood pressure; DDS: digital diabetes solution.
