# Supplementary material for: Clinical Effects of a Digital Health Intervention for Adults With Type 2 Diabetes in the United States: Retrospective Cohort Study
Source: J Med Internet Res. 2026 Jun 9;28:e66911. doi: 10.2196/66911 (PMC13291732; doi:10.2196/66911)
Supplement: Multimedia Appendix 9 [file jmir_v28i1e66911_app9.docx]

**Clinical Effects of a Digital Health Application in Patients with Type 2 Diabetes in the United States: A Retrospective Cohort Study**

**Multimedia Appendix 9**

**Table S1. Change in laboratory values from baseline.**

|  |  |  |  | Baseline^a^ | | Follow-up^b^ | | Difference-in-difference | *P* value |
| --- | --- | --- | --- | --- | --- | --- | --- | --- | --- |
|  |  |  |  | DDS users | DDS non-users | DDS users | DDS non-users |  |  |
|  | | | | | | | | | |
| **EMR measurement – blood glucose** | | | | | | | | | |
|  | N | | | 78 | 193 | 78 | 193 |  |  |
|  | % of patients | | | 13.7 | 11.4 | 13.7 | 11.4 |  |  |
|  | Mean (mg/dL) | | | 201.99 | 200.36 | 169.65 | 178.83 |  |  |
|  | SD (mg/dL) | | | 72.94 | 94.73 | 69.65 | 87.97 |  |  |
|  | Change from baseline^c^ (mg/dL) | | |  |  |  |  | –10.81 | .11 |
| **EMR measurement – total cholesterol** | | | | | | | | | |
|  | N | | | 52 | 125 | 52 | 125 |  |  |
|  | % of patients | | | 9.2 | 7.4 | 9.2 | 7.4 |  |  |
|  | Mean (mg/dL) | | | 174.27 | 171.02 | 171.35 | 160.98 |  |  |
|  | SD (mg/dL) | | | 56.66 | 45.20 | 52.47 | 43.86 |  |  |
|  | Change from baseline^c^ (mg/dL) | | |  |  |  |  | 7.12 | .76 |
| **EMR measurement – triglycerides** | | | | | | | | | |
|  | N | | | 55 | 122 | 55 | 122 |  |  |
|  | % of patients | | | 9.7 | 7.2 | 9.7 | 7.2 |  |  |
|  | Mean (mg/dL) | | | 197.55 | 199.12 | 188.93 | 186.17 |  |  |
|  | SD (mg/dL) | | | 98.07 | 131.59 | 142.36 | 124.95 |  |  |
|  | Change from baseline^c^ (mg/dL) | | |  |  |  |  | 4.33 | .61 |
| **EMR measurement – LCL-c** | | | | | | | | | |
|  | N | | | 52 | 120 | 52 | 120 |  |  |
|  | % of patients | | | 9.2 | 7.1 | 9.2 | 7.1 |  |  |
|  | Mean (mg/dL) | | | 98.79 | 92.55 | 92.56 | 85.4 |  |  |
|  | SD (mg/dL) | | | 47.88 | 35.66 | 40.75 | 32.54 |  |  |
|  | Change from baseline^c^ (mg/dL) | | |  |  |  |  | 0.92 | .82 |
| **EMR measurement – eGFR** | | | | | | | | | |
|  | N | | | 57 | 148 | 57 | 148 |  |  |
|  | % of patients | | | 10.0 | 8.7 | 10.0 | 8.7 |  |  |
|  | Mean (mL/min/1.73m^2^) | | | 89.68 | 91.36 | 89.03 | 89.75 |  |  |
|  | SD (mL/min/1.73m^2^) | | | 27.31 | 30.34 | 27.53 | 29.81 |  |  |
|  | Change from baseline^c^ (mL/min/1.73m^2^) | | |  |  |  |  | 0.96 | .63 |
| **EMR measurement – SBP** | | | | | | | | | |
|  | N | | | 111 | 325 | 111 | 325 |  |  |
|  | % of patients | | | 19.5 | 19.1 | 19.5 | 19.1 |  |  |
|  | Mean (mmHg) | | | 130.09 | 130.86 | 128.47 | 131.40 |  |  |
|  | SD (mmHg) | | | 14.13 | 15.93 | 13.88 | 15.86 |  |  |
|  | Change from baseline^c^ (mmHg) | | |  |  |  |  | –2.16 | .45 |
| **EMR measurement – DBP** | | | | | | | | | |
|  | N | | | 111 | 325 | 111 | 325 |  |  |
|  | % of patients | | | 19.5 | 19.1 | 19.5 | 19.1 |  |  |
|  | Mean (mmHg) | | | 78.43 | 77.51 | 77.22 | 77.43 |  |  |
|  | SD (mmHg) | | | 10.12 | 9.63 | 10.47 | 10.34 |  |  |
|  | Change from baseline^c^ (mmHg) | | |  |  |  |  | –1.13 | .35 |
| **EMR measurement – average SBP** | | | | | | | | | |
|  | N | | | 111 | 325 | 111 | 325 |  |  |
|  | % of patients | | | 19.5 | 19.1 | 19.5 | 19.1 |  |  |
|  | Mean (mmHg) | | | 129.50 | 130.76 | 128.95 | 131.43 |  |  |
|  | SD (mmHg) | | | 11.28 | 12.75 | 11.08 | 13.96 |  |  |
|  | Change from baseline^c^ (mmHg) | | |  |  |  |  | –1.22 | .55 |
| **EMR measurement – average DBP** | | | | | | | | | |
|  | N | | | 111 | 325 | 111 | 325 |  |  |
|  | % of patients | | | 19.5 | 19.1 | 19.5 | 19.1 |  |  |
|  | Mean (mmHg) | | | 78.4 | 78.0 | 77.3 | 77.4 |  |  |
|  | SD (mmHg) | | | 8.25 | 7.98 | 8.31 | 8.70 |  |  |
|  | Change from baseline^c^ (mmHg) | | |  |  |  |  | –0.48 | .30 |
| **EMR measurement** – **weight** | | | | | | | | | |
|  | N | | | 112 | 327 | 112 | 327 |  |  |
|  | % of patients | | | 19.7 | 19.3 | 19.7 | 19.3 |  |  |
|  | Mean (lbs) | | | 224.89 | 219.27 | 223.18 | 219.17 |  |  |
|  | SD (lbs) | | | 47.39 | 50.03 | 47.04 | 48.93 |  |  |
|  | Change from baseline^c^ (lbs) | | |  |  |  |  | –1.61 | .80 |
| **EMR measurement – BMI** | | | | | | | | | |
|  | N | | | 111 | 318 | 111 | 318 |  |  |
|  | % of patients | | | 19.5 | 18.7 | 19.5 | 18.7 |  |  |
|  | Mean (kg/m^2^) | | | 34.64 | 34.11 | 34.43 | 34.14 |  |  |
|  | SD (kg/m^2^) | | | 6.16 | 6.38 | 6.19 | 6.35 |  |  |
|  | Change from baseline^c^ (kg/m^2^) | | |  |  |  |  | –0.24 | .92 |

^a^ Highest value on the last date.

^a^ Highest value on the date closest to the index date.

^c^ Difference-in-difference is defined as DDS user change minus non-user change. *P*-value uses nonparametric Wilcoxon rank test to compare change of measurement (follow-up minus baseline) for each patient between DDS users and non-users.

BP: blood pressure; DBP: diastolic blood pressure; DDS: digital diabetes solution; eGFR: estimated glomerular filtration rate; EMR: electronic medical records; LDL-c: low-density lipoprotein cholesterol; SBP: systolic blood pressure.
