# Supplementary material for: Clinical Effects of a Digital Health Intervention for Adults With Type 2 Diabetes in the United States: Retrospective Cohort Study
Source: J Med Internet Res. 2026 Jun 9;28:e66911. doi: 10.2196/66911 (PMC13291732; doi:10.2196/66911)
Supplement: Multimedia Appendix 10 [file jmir_v28i1e66911_app10.docx]

**Clinical Effects of a Digital Health Application in Patients with Type 2 Diabetes in the United States: A Retrospective Cohort Study**

**Multimedia Appendix 10**

**Table S1. Change in CVD risk score.**

|  | | DDS users | DDS non-users | DDS users | DDS non-users | *P* value for baseline |
| --- | --- | --- | --- | --- | --- | --- |
|  | | | | | | |
| **Baseline** | | | | | | |
|  | Patients, n | 87 | 245 |  |  |  |
|  | Mean for Baseline: Index Date –365 days to Index Date +30 days |  |  | 16.49 | 16.98 | .32^a^ |
|  | Low FRS <10%, n (%) |  |  | 11 (12.6) | 30 (12.2) | .23^a^ |
|  | Intermediate FRS 10% to <20%, n (%) |  |  | 30 (34.5) | 62 (25.3) |  |
|  | High FRS ≥20%, n (%) |  |  | 46 (52.9) | 153 (62.5) |  |
| **12-month follow-up** | | | | | | |
|  | Patients, n | 87 | 250 |  |  |  |
|  | Mean for Baseline: Index Date –365 days to Index Date +30 days |  |  | 16.39 | 17.02 | .25^a^ |
|  | Low FRS <10%, n (%) |  |  | 13 (14.9) | 26 (10.4) | .52^a^ |
|  | Intermediate FRS 10% to <20%, n (%) |  |  | 12 (26.4) | 70 (28.0) |  |
|  | High FRS ≥20%, n (%) |  |  | 51 (59.6) | 154 (61.6) |  |

^a^ CVD score calculation method.

CVD: cardiovascular disease; DDS: digital diabetes solution; FRS: Framingham Risk Score.
